# Diversity of Picorna-Like Viruses in the Teltow Canal, Berlin, Germany

**DOI:** 10.3390/v16071020

**Published:** 2024-06-25

**Authors:** Roland Zell, Marco Groth, Lukas Selinka, Hans-Christoph Selinka

**Affiliations:** 1 Section of Experimental Virology, Institute for Medical Microbiology, Jena University Hospital, Friedrich Schiller University, 07743 Jena, Germany; 2 CF Next Generation Sequencing, Leibniz Institute on Aging-Fritz Lipmann Institute, 07745 Jena, Germany; 3 Section II 1.4 Microbiological Risks, Department of Environmental Hygiene, German Environment Agency, 14195 Berlin, Germany

**Keywords:** riverine ecosystems, viromes, Picornavirales, phylogenetic analysis, metagenomic, picorna-like viruses, RNA viruses

## Abstract

The viromes of freshwater bodies are underexplored. The *Picornavirales* order, with 371 acknowledged species, is one of the most expansive and diverse groups of eukaryotic RNA viruses. In this study, we add 513 picorna-like viruses to the assemblage of more than 2000 unassigned picorna-like viruses. Our set of the aquatic *Picornavirales* virome of the Teltow Canal in Berlin, Germany, consists of 239 complete and 274 partial genomes. This urban freshwater body is characterized by the predominance of marna-like viruses (30.8%) and dicistro-like viruses (19.1%), whereas picornaviruses, iflaviruses, solinvi-like viruses, polycipi-like viruses, and nora-like viruses are considerably less prevalent. Caliciviruses and secoviruses were absent in our sample. Although presenting characteristic domains of *Picornavirales*, more than 100 viruses (20.8%) could not be assigned to any of the 9 *Picornavirales* families. Thirty-three viruses of the *Marnaviridae*—mostly locarna-like viruses—exhibit a monocistronic genome layout. Besides a wealth of novel virus sequences, viruses with peculiar features are reported. Among these is a clade of untypeable marna-like viruses with dicistronic genomes, but with the capsid protein-encoding open reading frame located at the 5′ part of their RNA. A virus with a similar genome layout but clustering with dicistroviruses was also observed. We further detected monocistronic viruses with a polymerase gene related to aparaviruses. The detection of Aichi virus and five novel posa-like viruses indicates a slight burden in municipal wastewater.

## 1. Introduction

The *Picornavirales* order comprises nine virus families with characteristic features (hereafter called picorna-like viruses, PLVs), i.e., *Caliciviridae*, *Dicistroviridae*, *Iflaviridae*, *Marnaviridae*, *Noraviridae*, *Picornaviridae*, *Polycipiviridae*, *Secoviridae*, and *Solinviviridae* [1]. Members of these families have a non-enveloped icosahedral capsid with a T = 1/pseudo-T = 3 or T = 3 structure. Genomic RNA is polyadenylated and supposed to be covalently linked at its 5’-end with a small, virus-encoded polypeptide called VPg (viral protein genome-associated), but experimental confirmation is lacking for some viruses. The genome layouts of the various taxa are highly variable. All members of *Picornavirales* share phylogenetically conserved helicase (hel), proteinase (pro), and polymerase (pol) domains as well as one to three capsid proteins (CP) with a jellyroll fold. The three conserved non-structural proteins are encoded by the so-called “hel-pro-pol core replicative module” or “hel-pro-pol replication block” [2,3], which is a characteristic of all members of the *Picornavirales* order. The helicase has two conserved sequence elements of P-loop ATPases, the Walker A (GxxGxGKS/T) and B motifs (DD or DE). The proteinase has a chymotrypsin-like fold and a H-D/E-C/S catalytic triad with the nucleophile in a GxCG or GxSG active-site sequence context. The RNA-dependent RNA polymerase (RdRp) has conserved amino acids of the active site, e.g., the DxxxxD, GxxxTxxxN, and GDD motifs. Supplementary Figure S1 summarizes the various genome layouts found in members of the *Picornavirales* order. The RdRp is the only protein that is conserved in all RNA viruses. Therefore, this universal protein has been used for virus taxonomy. A global RdRp phylogenetic tree inferred by Wolf et al. (2018) is composed of five branches corresponding to the newly created virus phyla, with the members of *Picornavirales* clustering on branch 2 (phylum *Pisuviricota*) [4]. In addition to the acknowledged viruses of *Picornavirales*, at least two thousand PLVs with hallmarks of the *Picornavirales* order have been described in recent years and await formal classification [5,6,7,8,9,10,11]. However, little sequence similarity renders the accommodation of most of these new viruses to existing virus taxa difficult and suggests the existence of many new virus species, genera, and families. This diversity—fascinating and dazing at the same time—is one constraint in the creation of an adequate up-to-date *Picornavirales* taxonomy.

The host range of the members of *Picornavirales* and unclassified PLVs is rather broad. It includes vertebrates (*Picornaviridae* and *Caliciviridae*); arthropods like insects, arachnids, crustaceans, and myriapods (*Dicistroviridae*, *Iflaviridae*, *Noraviridae*, *Polycipiviridae*, and *Solinviviridae*); higher plants (*Secoviridae*); and unicellular algae and protists like diatoms, raphidophytes, and thraustochytrids (*Marnaviridae*). Furthermore, many unclassified PVLs have been detected in the organ/tissue samples of annelids, nematodes, mollusks, echinoderms, anthozoans, seaweed, and terrestrial higher plants, but also in fecal samples of various organisms and in environmental substrates like freshwater, sea water, wastewater, soil, and sediment. The repeated detection of very similar PLVs in various samples and locations suggests a wide distribution, at least of some PLVs. It has been argued that PVLs may accumulate in many organisms after food uptake rather than infect them, an assumption that contributes to the confusion about the PLV host range. A lack of knowledge of hosts hampers research, as virus isolation and propagation in suitable cell lines or hosts is critical for the investigation of the respective viruses. Many members of the order *Picornavirales* are proven pathogens, but an increasing number of recent metagenomic studies revealed numerous apparently non-virulent viruses. Their ecological role is still insufficiently studied.

Culture-independent virus sequencing facilitated the identification of a plethora of new viruses. Among the most expansive and diverse groups of eukaryotic RNA viruses is the *Picornavirales* order, with 371 acknowledged species and more than 2000 unassigned PLVs. The majority of metagenomic studies, however, focused on marine ecosystems, while the viromes of freshwater bodies remained underexplored [12,13,14,15]. In order to understand the virus diversity detected in two German rivers, we analyzed the RNA viromes of the Havel River and the Teltow Canal in Berlin and published partial results [8,16,17]. Here, we present the sequences of more than 500 PLVs detected in the Teltow Canal (named TC-PLVs), many of which are novel, with little similarity to known members of the *Picornavirales*. Our study contributes to closing a knowledge gap and providing an answer to the question of whether the abundant preponderance of marine PLVs reflects the natural situation or results from a distorted record.

## 2. Materials and Methods

### 2.1. Sample Collection and Workup

The viruses in this study were enriched from a 50 L water sample (sample ID MR233-17) collected in Berlin, Bäkebrücke (site coordinates: 52°26′03″ N, 13°18′57″ E), on 18 July 2017. Details of the enrichment procedure were described previously [8,16,17]. Briefly, five 10 L aliquots were vigorously stirred for 20 min, and the pH was adjusted from 8.1 to 3.5. Virus particles were adsorbed to glass wool according to Wyn-Jones et al. [18] and eluted with 3% beef extract/0.05 M glycine buffer pH 9.5. Thereafter, the pH of the eluate was adjusted to 7. Residual detritus and bacteria of the eluate were removed by filtering (0.45 µm). Virus particles were sedimented using ultracentrifugation (100,000× *g*, 2.5 h at 4 °C). The sediment was redissolved in 0.5 mL phosphate-buffered saline and homogenized using a ball mill. The virus suspension was stored at −80 °C. RNA extraction was performed with the QIAamp Viral RNA mini kit (Qiagen, Hilden, Germany), according to the manufacturer’s instruction.

### 2.2. Illumina Sequencing, Sequence Data Processing, and Virus Sequence Analyses

For Illumina sequencing, a library was prepared with 450 ng of total RNA using Illumina’s TruSeq stranded total RNA library preparation kit combined with the Ribo-Zero Gold rRNA Removal Kit. The library was quantified and quality checked utilizing the 2100 Bioanalyzer and a DNA 7500 assay. Sequencing (2 × 150 bp paired-end) was performed on a HiSeq 2500 platform using the rapid run mode. Then, the sequence data were extracted in FastQ format using bcl2FastQ v2.19.1.403 (Illumina), and adapter sequences were removed with Cutadapt v1.8.3 software [19] as described previously [17]. After adapter trimming and the removal of duplicons, 70,018,635 read pairs were used for de novo assembly with clc_assembler v5.2.1 (Qiagen) using the parameters -p fb ss 50 500 and metaSPAdes v3.15.3 [20] using standard parameters (-k auto). The clc_assembler yielded 537,529 contigs greater than 200 nucleotides, and metaSPAdes yielded 1,314,849 scaffolds. In order to obtain the final sequences, some sequences were manually curated by connecting overlapping contigs/scaffolds if appropriate.

For a first sequence data analysis, all contigs from the clc assembler and scaffolds from metaSPAdes were used to search a virus protein database compiled from all NCBI GenBank entries with the Taxonomy ID 10239 using DIAMOND v2.0.10 [21]. Specific searches were executed with BLAST+ v2.13.0 (https://ftp.ncbi.nlm.nih.gov/blast/executables/blast+/2.13.0/, accessed on 5 June 2024) using the BLASTp, tBLASTx, and BLASTn tools as well as reference sequences downloaded from GenBank. Protein domains were predicted using the NCBI web search tools BLASTp suite (https://blast.ncbi.nlm.nih.gov/Blast.cgi, accessed on 5 June 2024) and the Pfam conserved domain database (CDD; https://www.ncbi.nlm.nih.gov/Structure/cdd/wrpsb.cgi, accessed on 5 June 2024). Sequence alignments were conducted with ClustalW or Muscle, programs implemented in Mega version X [22]. Phylogenetic analyses were inferred with IQ-TREE 2.1.3 for Windows [23] utilizing the automatic model selection option (ModelFinder) of IQ-TREE for the identification of the best-fit substitution models. Branch support was assessed using standard non-parametric bootstrap analysis (1000 replications) for simple datasets or the ultrafast bootstrap approximation UFBoot2 (10,000 replications) for complex alignments [24].

For virus assignment, the current virus taxonomy (2023 release) based on the most recent Master Species List #39 was used (https://ictv.global/msl; accessed on 20 June 2024).

## 3. Results

### 3.1. Sequencing, Assembly, and Identification of Viral Sequences

**Figure 1:**
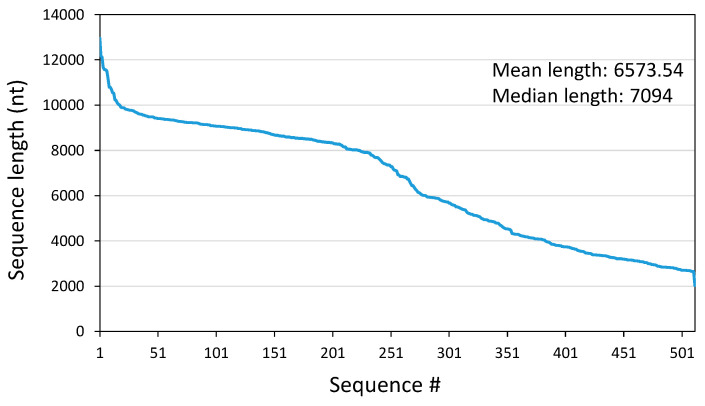
Sequences of 513 Teltow Canal picorna-like viruses sorted by length.

Illumina paired-end sequencing resulted in 70,018,635 read pairs that were assembled to 1,314,849 scaffolds using metaSPAdes and 537,529 contigs using clc assembler (with a length ≥200 nt). DIAMOND identified 66,371 virus-specific scaffolds and 41,408 contigs with e-values <10^−10^, of which 7386 scaffolds/5587 contigs were classified as sequences of the order *Picornavirales*. In a second step, the largest contigs were subjected to a BLASTp search in order to confirm the DIAMOND results. In most cases, the DIAMOND classification to the *Picornavirales* order was confirmed using this approach, but the assignments to the taxonomic levels family, genus, and species were wrong. Despite the identification of thousands of *Picornavirales* sequences, we focused on 513 of the largest PLV sequences, with sizes ranging from 12,961 to 2057 nt (mean length, 6573.54 nt; median length, 7094 nt). Figure 1 presents the size distribution of these sequences. Twelve of these sequences were assigned to the genera *Ampivirus* (*Picornaviridae*), *Kobuvirus* (*Picornaviridae*), *Chipolycivirus* (*Polycipiviridae*), *Cripavirus* (*Dicistroviridae*), and *Iflavirus* (*Iflaviridae*). The remaining 501 sequences were novel, but about 130 TC-PLVs exhibited similarity to previously described unclassified PLVs. The coverages of our sequences varied considerably; the mean depths ranged from 7.27 to 90,933.2 (Table S1).

### 3.2. Phylogenetic Analyses

**Figure 2:**
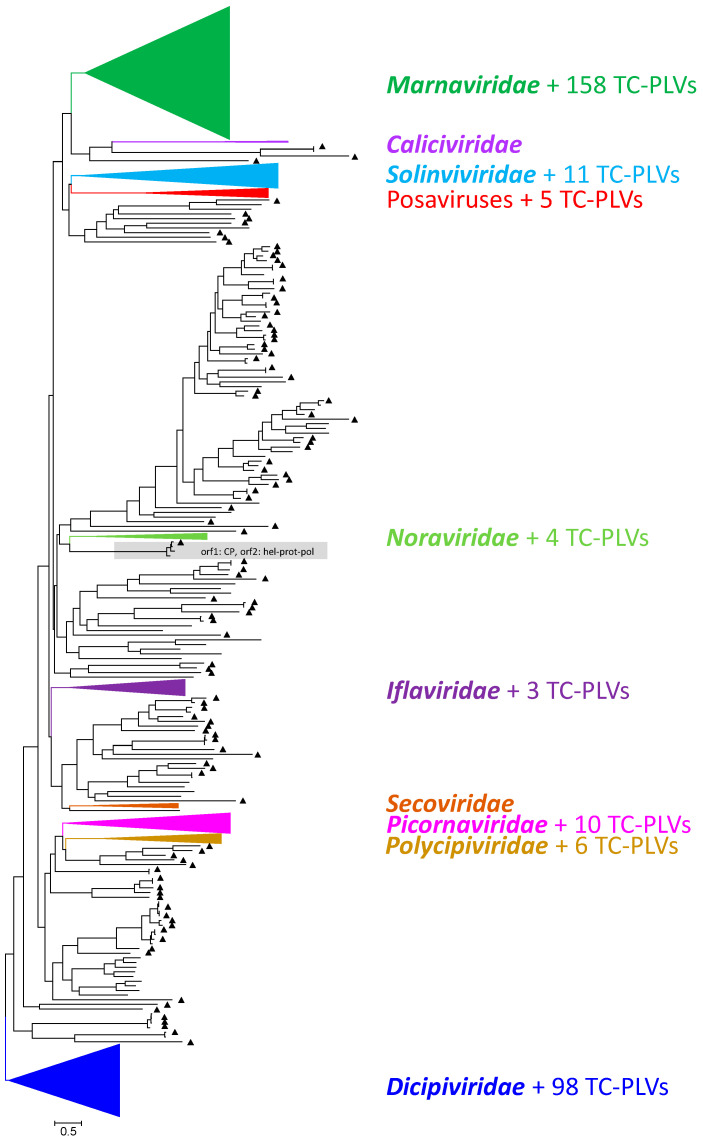
Phylogenetic analysis of RdRp sequences. A total of 934 RdRp amino acid sequences representing all nine families of the *Picornavirales* order plus numerous unclassified viruses were aligned with the help of ClustalW. The alignment comprises 111 classified viruses, 398 viruses from the Teltow Canal, 100 viruses from the Havel River in Berlin, and 330 unclassified virus sequences downloaded from GenBank. The maximum likelihood tree was inferred with IQ-TREE 2.1.3 using the Q.pfam+F+R9 substitution model. Ultrafast bootstrap support was obtained with 10,000 replications. Branches with members of the nine *Picornavirales* families are condensed for better comprehensibility. Each family is indicated by a different color. Untypeable sequences are printed in black. Filled triangles (▲) indicate PLVs from the Teltow Canal. The bar indicates the number of substitutions per site. The tree was arbitrarily rooted with the clade of dicistro-like viruses. Details of the tree are presented in Figure S2.

As the replicase is the only universal conserved protein of all RNA viruses [4], we used an RdRp alignment for the preliminary assignment of our sequences to the known families of the *Picornavirales*. Our alignment comprised 934 sequences, i.e., 111 recognized reference strains representing the 9 families of the *Picornavirales* and 823 unclassified PLVs (393 sequences from the Teltow Canal sample, 100 Havel picorna-like virus sequences [8], and 330 sequences downloaded from GenBank). The tree was inferred with IQ-TREE 2.1.3. A simplified version of the unrooted tree is shown in Figure 2, whereas its details are presented in Supplementary Figure S2. On the basis of the RdRp tree and the BLASTp results, 158 TC-PLVs were preliminarily assigned to the *Marnaviridae* family, 98 TC-PLVs were dicistrovirus-like, 11 solinvivirus-like, 4 noravirus-like, 6 polycipivirus-like; 3 TC-PLVs clustered with iflaviruses, and 10 with picornaviruses (Table 1). None of the TC-PLVs grouped with sequences of *Caliciviridae* and *Secoviridae*, and 106 sequences remained untypeable. These untypeable TC-PLVs segregate into several distinct branches, suggesting the existence of yet-undefined virus taxa, one of which is a clade consisting of posaviruses, posa-related viruses and five TC-PLVs.

#### 3.2.1. *Marnaviridae* and Related Viruses

The *Marnaviridae* family is presently composed of seven genera (*Bacillarnavirus*, *Kusarnavirus*, *Labyrnavirus*, *Locarnavirus*, *Marnavirus*, *Salisharnavirus*, and *Sogarnavirus*), with twenty species altogether [25]. Additionally, the *Marnaviridae* Study Group has provisionally assigned 135 virus sequences from metagenomic studies to these genera (https://ictv.global/report/chapter/marnaviridae/marnaviridae, accessed on 12 May 2024), but hundreds of unclassified sequences related to the *Marnaviridae* have been deposited in the GenBank in the meantime. Monocistronic and dicistronic gene layouts have been described with the nonstructural polyprotein-encoding sequence in the 5′-region of the genome and the capsid polyprotein-encoding sequence in the 3′-region. Heterosigma akashiwo RNA virus (genus *Marnavirus*), marine RNA virus SF-3 (genus *Locarnavirus*), plus a few unclassified viruses have a monocistronic genome. The remaining members of the family have dicistronic genomes. Aurantiochytrium single-stranded RNA virus (*Labyrnavirus*) is exceptional in that its RNA contains a third orf that is transcribed as a subgenomic RNA [26]. Whether other kusarnaviruses exhibit this feature is unknown. All classified marnaviruses have been isolated from marine protists or were detected in marine environmental samples.

The *Marnaviridae* branch of our RdRp tree comprises classified and related viruses and contains 334 sequences. The branch segregates in many subclades, partly with long branch lengths. This indicates substitution saturation and suggests the existence of novel taxa. Apparantly, bacillarnaviruses, kusarnaviruses, locarnaviruses, salisharnaviruses, and sogarnaviruses cluster monophyletically, whereas labyrnaviruses and marnaviruses, which are located at the root of the *Marnaviridae* branch, do not. At least 106 of the TC-PLVs cluster with the locarnaviruses (Table 1), 10 with the labyrnaviruses, 8 with the salisharnaviruses, and 11 with the sogarnaviruses. No TC-PLVs group with the bacillarna- and kusarnaviruses. Twenty TC-PLVs are difficult to assign to an acknowledged genus of the *Marnaviridae* family and remain untyped (Table 1). Altogether, 59 viruses with monocistronic genomes were identified, 33 of which are TC-PLVs (Table S1) and 7 HPLVs. They are scattered on the *Marnaviridae* branch: among them are 29 locarnavirus candidates, 1 marnavirus candidate and 3 untyped TC-PLVs. Interestingly, a clade of seven viruses, including sequences from the Teltow Canal, Havel River, and the Manatee freshwater spring in Florida, exhibits an unusual bicistronic genome layout, as orf 1 encodes the capsid proteins, and orf 2 codes for the nonstructural polyprotein.

#### 3.2.2. *Dicistroviridae* and Related Viruses

Members of the family *Dicistroviridae* have a dicistronic genome with a 5′-orf-encoding helicase, proteinase, and RdRp and a capsid protein-encoding 3′-orf. The 5′-end of genomic RNA is covalently linked to a VPg protein. Both orfs are separated by an intergenic region. The translation of both orfs is driven by two IRES sequences with characteristic sequences. The family comprises three genera (*Aparavirus*, *Cripavirus*, and *Triatovirus*) with sixteen species so far [27]. All dicistroviruses recognized by the ICTV infect arthropods. In recent times, however, numerous unclassified dicistro-like viruses from other sources than arthropods have been described, e.g., in samples of mollusks, anthozoans, flowering plants, algae, intestinal contents of mammals, cloacal swabs, fecal samples, soil, sediment, and environmental water.

The dicistrovirus branch of our RdRp tree included 14 sequences of recognized dicistrovirus species and 176 sequences of unclassified viruses. Among these were 98 TC-PLVs, 24 HPLVs, and 54 sequences downloaded from GenBank. The tree revealed 11 TC-PLVs that are distantly related to aparaviruses. Cripaviruses did not show monophyly, a finding that was also observed in a previous study [8]. Three cripaviruses from the Teltow Canal cluster with aphid lethal paralysis virus and Drosophila C virus and are supposed to be new strains of these viruses. Many clades suggest the existence of additional dicistrovirus taxa. Furthermore, of special interest is the unusual genome layout of TC-PLV-57: its orf 1 encodes the capsid proteins and orf 2 the nonstructural polyprotein. In addition, four PLVs have monocistronic genomes. Of these, only TC-PLV-154 and TC-PLV-201 cluster together.

#### 3.2.3. *Solinviviridae* and Related Viruses

**Figure 3:**
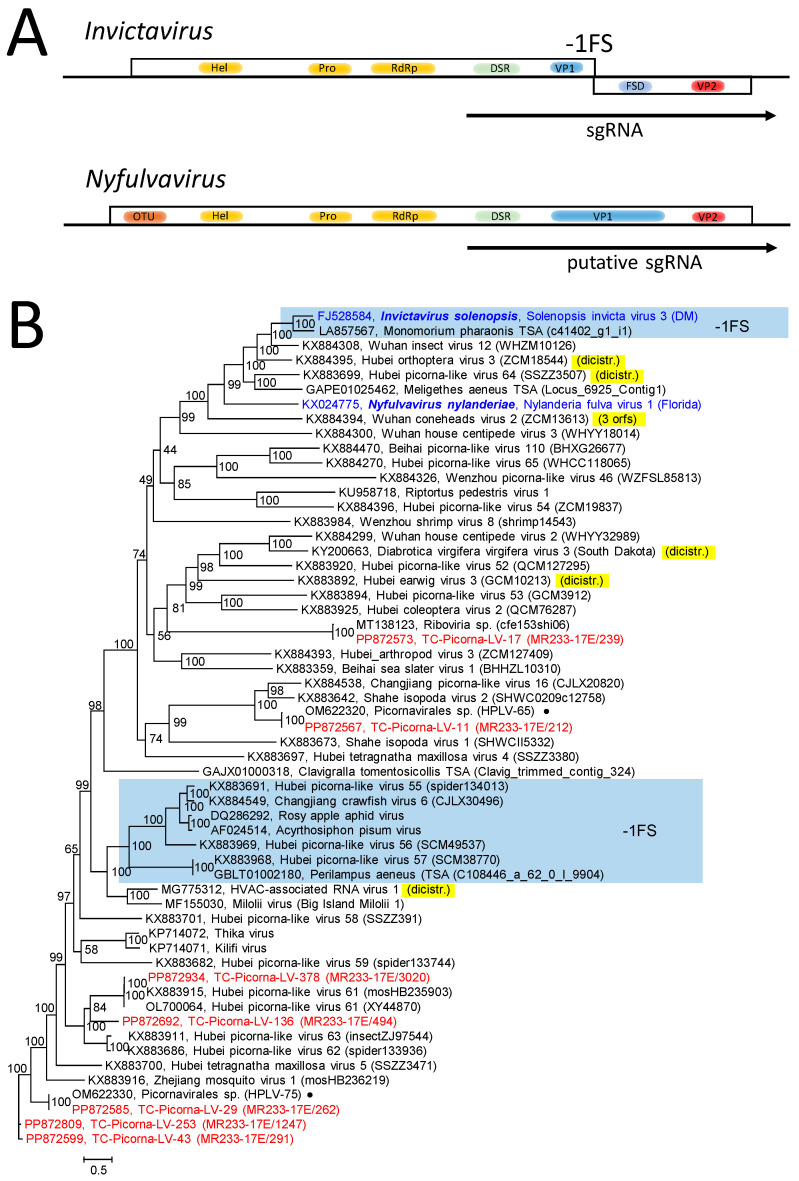
*Solinviviridae* and related viruses. (**A**) Genome organization of *Invictavirus* and *Nyfulvavirus*. Boxes represent orfs, and arrows indicate transcription of subgenomic RNA (sgRNA). (**B**) Phylogenetic analysis of hel-prot-RdRp-DSR-VP1-FSD gene region. Fifty-seven sequences of the solinvivirus clade of Figure 2 and Figure S2 were aligned and used for tree inference with IQ-TREE 2.1.3. Optimal substitution model: Q.pfam+F+R7. Presented is the unrooted maximum likelihood tree. Numbers at nodes indicate ultrafast bootstrap support obtained with 10,000 replications. The scale bar indicates the number of substitutions per site. Sequences with -1 frameshift are boxed and labeled with -1FS. Viruses with deviant di- or tricistronic genomes are marked in yellow. Color code: blue, classified reference viruses; red, Teltow Canal picorna-like viruses (TC-PLVs); black, unclassified viruses. Presented are GenBank accession numbers, species names (printed in bold and in italics), virus names, and strain designations/sequence identifiers (in round brackets). Dots (●) indicate PLVs from the Havel River. Abbreviations: DSR, dsRNA-binding protein; FSD, frameshift domain; hel, helicase; OTU, ovarian tumor domain; prot, proteinase; RdRp, RNA-dependent RNA polymerase; VP1, viral protein1; VP2, viral protein 2; HPLV, Havel picorna-like virus; TC-PLV, Teltow Canal picorna-like virus.

The *Solinviviridae* family presently consists of two genera, each with only one species [28]. The genome of Nylanderia fulva virus (genus *Nyfulvavirus*) consists of a single orf encoding a polyprotein with an ovarian tumor (OTU) domain, helicase, proteinase and RdRp domains, an dsRNA binding protein, a viral protein (VP) 1 with a jellyroll domain, and VP2 (Figure 3A). Polyprotein processing is facilitated with the viral proteinase. The genome of Solenopsis invicta virus 3 (genus *Invictavirus*) has two orfs (Figure 3A). Orf 1 codes for helicase, proteinase, RdRp, dsRNA binding protein (DSR), and VP1. The second orf is expressed from a subgenomic RNA (sgRNA). This sgRNA encodes the dsRNA binding protein and VP1 of orf 1 and a frameshift domain (FSD) and VP2 of orf 2. Via a -1 frameshift, a VP1-FSD and VP2 are produced. Based on RdRp sequence similarity, the *Solinviviridae* Study Group assumes an additional 65 related yet unclassified viruses belonging to the family (https://ictv.global/report/chapter/solinviviridae/solinviviridae, accessed on 12 May 2024).

The phylogenetic analysis of the RdRp sequence revealed a clade of 59 solinvi- and solinvi-like viruses, including 11 TC-PLVs and 2 HPLVs (Figure S1). All Teltow Canal solinvi-like viruses had a monocistronic genome layout with lengths ranging from 5669 nt (partial genome) to 10,226 nt. The result was confirmed with a phylogenetic analysis based on an alignment of sequences comprising the OTU–helicase–proteinase–polymerase–DSR–VP1 sequences (Figure 3B). This figure further indicated a clade with seven viruses that exhibited a second orf and a -1 frameshift signal. Furthermore, six viruses showed two or three orfs without a -1 frameshift signal.

#### 3.2.4. *Iflaviridae* and Related Viruses

The family *Iflaviridae* presently comprises a single genus, *Iflavirus*, with 16 species [29]. All iflaviruses infect arthropods. They have a monocistronic, polyadenylated genome with a covalently linked VPg at its 5’-end. Translation is driven by an IRES. A small leader protein and four capsid proteins with the order VP2, VP4, VP3, and VP1 are located at the N-terminus of the polyprotein, and the nonstructural proteins, including the helicase, proteinase, VPg, and RdRp domains, configure the C-terminal part.

Our RdRp tree comprised 13 acknowledged iflavirus species and 23 related, unclassified viruses, plus 3 viruses from the Teltow Canal sample and 1 virus from the Havel River. Whereas Teltow Canal iflavirus 2 was almost identical to Myrmica rubra picorna-like virus 3, Teltow Canal iflavirus 1/HPLV-14 and Teltow Canal iflavirus 3 likely represent novel iflavirus species.

#### 3.2.5. *Polycipiviridae* and Related Viruses

Polycipiviruses have rather long genomes, 10–12 kb in length, with five orfs [30]. Orfs 1, 3, and 4 encode capsid proteins with jellyroll folds, whereas the product of orf 2 has unknown function. The translation of these orfs is driven from an IRES located in the 5’-untranslated region and a postulated ribosome reinitiation mechanism. Orf 5 codes for a nonstructural polyprotein with helicase, proteinase, and RdRp domains and presumably for a VPg polypeptide. The translation of this orf is driven by a second IRES. Three polycipivirus genera have been described—*Chipolycivirus*, *Hupolycivirus*, and *Sopolycivirus*—with 14 species altogether [30]. The known polycipiviruses have been detected in insects and spiders.

**Figure 4:**
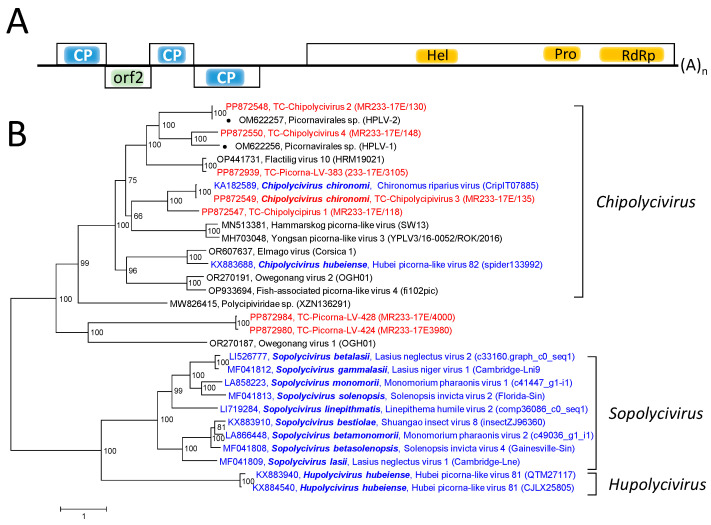
*Polycipiviridae* and related viruses. (**A**) Genome organization of polycipiviruses. Five orfs are indicated as boxes. The location of capsid proteins (CPs) with a jellyroll fold, helicase (hel), proteinase (prot), and RdRp is indicated. The function of the orf 2 gene product is unknown. (**B**) Phylogenetic analysis of five concatenated orfs of polycipiviruses. Thirty sequences of polycipiviruses and polycipi-like viruses were aligned with the help of ClustalW and used for tree inference with IQ-TREE 2.1.3. Optimal substitution model: Q.pfam+F+R5. Presented is the unrooted maximum likelihood tree. Numbers at nodes indicate ultrafast bootstrap support obtained with 10,000 replications. The scale bar indicates the number of substitutions per site. Color code: blue, classified reference viruses; red, Teltow Canal viruses (TC-Chipolycivirus, TC-PLV); black, unclassified viruses. Presented are GenBank accession numbers, species names (printed in bold and in italics), virus names, and strain designations/sequence identifiers (in round brackets). Square brackets indicate three polycipivirus genera. Abbreviations: CP, capsid protein; hel, helicase; orf, open reading frame; prot, proteinase; RdRp, RNA-dependent RNA polymerase; HPLV, Havel picorna-like virus; TC-PLV, Teltow Canal picorna-like virus.

The RdRp tree (Figure S1) includes eleven recognized sopolicivirus sequences, two chipoliciviruses, and one hupolicivirus (according to https://ictv.global/report/chapter/polycipiviridae/polycipiviridae, accessed on 5 June 2024). In addition, three chipolycivirus-like sequences downloaded from GenBank, six polycipivirus-like sequences from the Teltow Canal, and two from the Havel River were included. Four TC-PLVs with almost complete genomes exhibit similarity to chipolyciviruses, whereas two related sequences (TC-PLV-424 and TC-PLV-428) show only little similarity to the three known polycipivirus genera and likely belong to a novel genus in the *Polycipiviridae* family. In order to confirm this result, concatenated sequences including all five orfs were aligned and used to infer a phylogenetic tree. Besides TC-PLV-424 and TC-PLV-428, Owegonang virus 1 also exhibited a remarkable sequence divergence, as indicated by long branch lengths (Figure 4).

#### 3.2.6. *Picornaviridae* and Related Viruses

The *Picornaviridae* family, one of the most expansive RNA virus families, presently comprises 5 subfamilies, 68 genera, and 158 species [31,32]. Genome sizes range between 7 and 10.2 kb. Viral RNA has positive strand polarity, is polyadenylated, and contains a virus-encoded peptide (3B, VPg) covalently linked to the 5’-end. The genome layout is variable between the genera, but with exception of the dicipiviruses, all picornaviruses have a monocistronic genome encoding a large polyprotein with capsid proteins at its N-terminal part and nonstructural proteins at the C-terminal part. Some picornaviruses have a leader protein preceding the structural proteins. Three or four capsid proteins are found in the order 1A(VP4)-1B(VP2)-1C(VP3)-1D(VP1) or 1AB(VP0)-1C(VP3)-1D(VP1). At least seven nonstructural proteins (2A, 2B, 2C, 3A, 3B, 3C, and 3D) have been described, of which only proteins 2C (helicase), 3C (proteinase), and 3D (RdRp) are conserved in this family.

**Figure 5:**
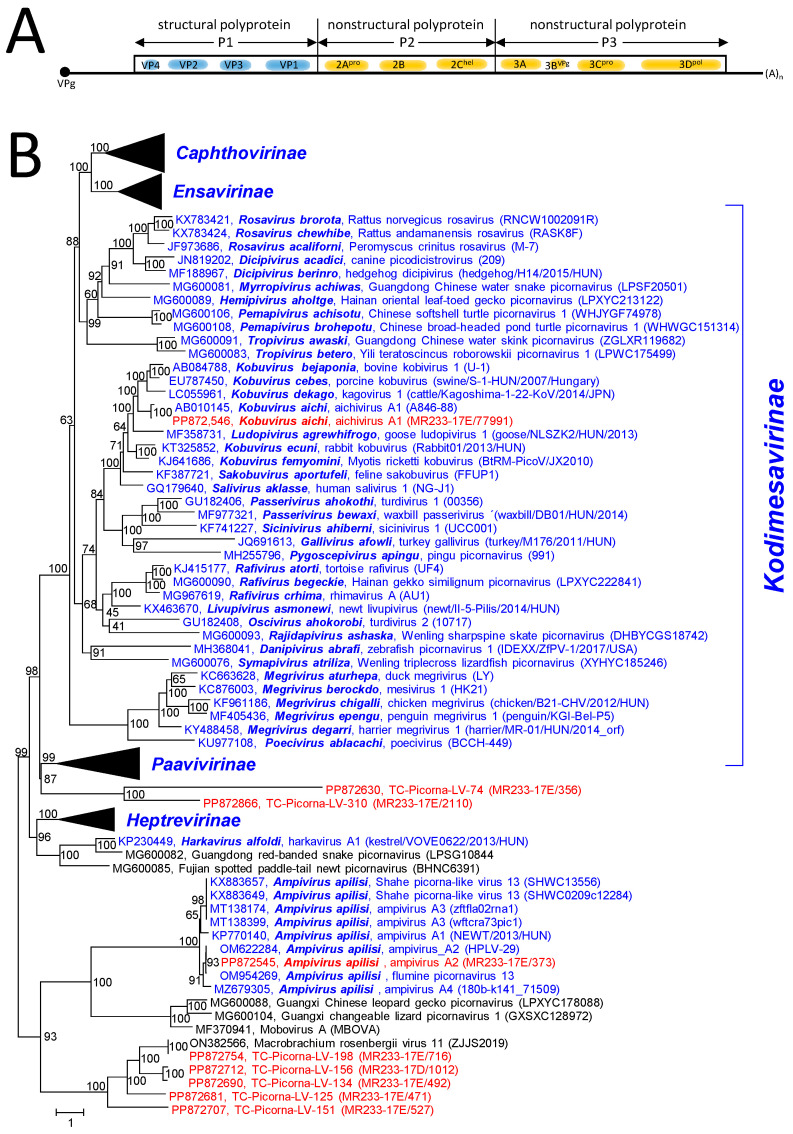
*Picornaviridae* and related viruses. (**A**) Genome organization of paradigmatic poliovirus. The single orf is indicated as a box. The locations of capsid proteins (VP1 to VP4) and nonstructural proteins are indicated. (**B**) Phylogenetic analysis of capsid protein sequences. A total of 164 sequences of picornavirus species, 9 Teltow Canal viruses (TC-PLVs), 1 Havel virus (HPLV), and 6 unclassified picornaviruses were aligned with the help of ClustalW and used for tree inference with IQ-TREE 2.1.3. Optimal substitution model: Q.pfam + F + R7. Presented is the maximum likelihood tree. Numbers at nodes indicate ultrafast bootstrap support obtained with 10,000 replications. The scale bar indicates the number of substitutions per site. Color code: blue, classified reference viruses; red, Teltow Canal viruses; black, unclassified viruses. Presented are GenBank accession numbers, species names (printed in bold and in italics), virus names, and strain designations/sequence identifiers (in round brackets). Branches of the monophyletic subfamilies *Caphthovirinae*, *Ensavirinae*, *Heptrevirinae*, and *Paavivirinae* are condensed. The square bracket indicates the members of the *Kodimesavirinae* subfamily, which is not monophyletic. The tree is arbitrarily rooted with the highly diverse clade of ampiviruses and other yet-unclassified picornavirus candidates. Details of the tree are presented in Figure S3.

The RdRp tree (Figure S2) includes 33 picornavirus reference sequences (31 sequences of the 5 acknowledged subfamilies plus harkavirus and ampivirus sequences, which have not yet been assigned to a subfamily). Ampivirus-like sequences downloaded from GenBank and an ampivirus sequence from the Havel River (HPLV-29) were also included. The resulting RdRp tree indicated the presence of Aichi virus and ampivirus in the Teltow Canal sample. Furthermore, eight virus sequences—all with picornavirus hallmarks—cluster with the ampiviruses and may represent new picornaviruses. In order to address this question, the polyprotein P1 (CP) sequences of 180 picornaviruses, which represent all acknowledged picornavirus species plus several picornavirus candidates, were aligned and analyzed. The resulting tree confirms the presence of Aichi virus in the Teltow Canal and indicates the split of ampiviruses into four (geno-)types. As was observed previously, the members of *Kodimesavirinae* do not show monophyly in the CP tree (https://ictv.global/report/chapter/picornaviridae/picornaviridae, accessed on 12 May 2024). In addition, the P1 tree shows a clade of six picornavirus candidates grouping with ampiviruses and mobovirus and another branch with two picornavirus candidates from the Teltow canal clustering close to the *Heptrevirinae* (Figure 5 and Figure S3).

#### 3.2.7. *Noraviridae* and Related Viruses

The *Noraviridae* family is the youngest family in the *Picornavirales* order. It includes a single genus, *Orthonoravirus*, with only one species, but a number of nora-related viruses have been described, e.g., [5,8,10,11,33,34]. While the acknowledged orthonoraviruses have been detected in samples of fruit flies (*Drosophila spec.*) and honeybees (*Apis mellifera*), nora-related viruses are associated with various arthropods like insects, spiders, crustaceans (crabs, crayfish, and shrimp), and woodlice, but also with sea anemones, mollusks (mussels, snails, and octopus), and environmental samples (rivers and sediment from freshwater bodies). Orthonoraviruses have a genome layout with four orfs. Their products are named VP1 to VP4, with VP2 and VP4 being polyproteins that are processed by a virus-encoded proteinase to yield mature nonstructural proteins including helicase, proteinase, and RdRp, and three CPs (VP4A, VP4B, and VP4C) [33,34].

**Figure 6:**
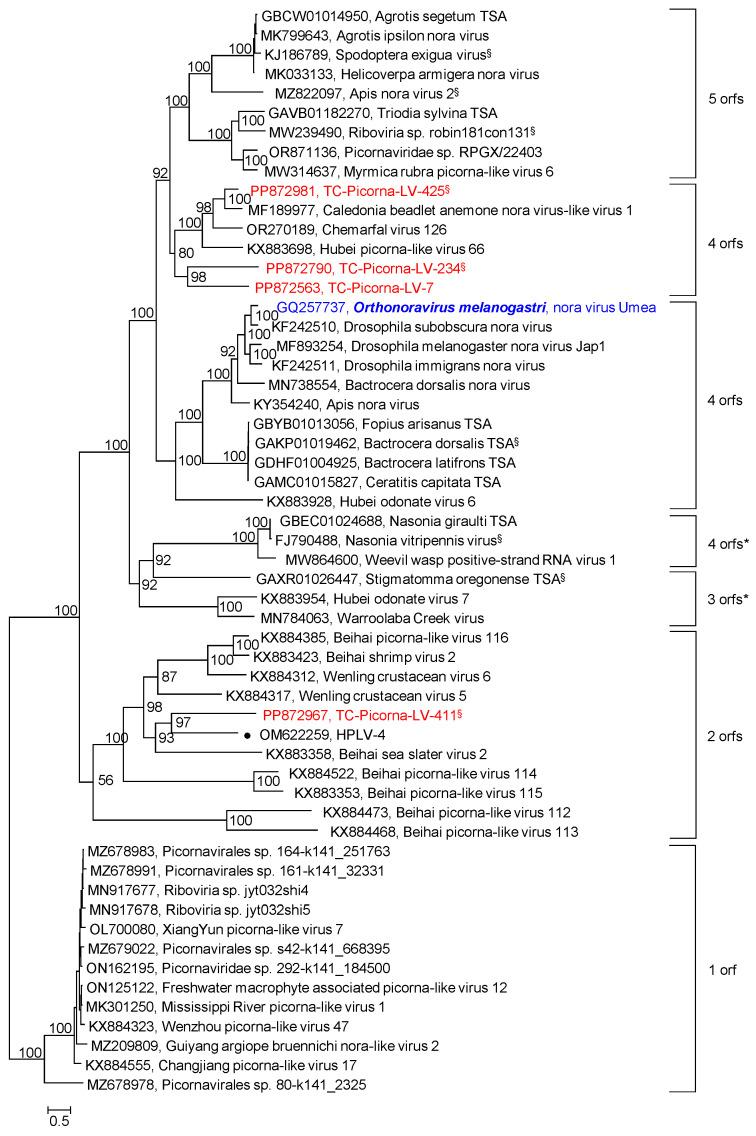
*Noraviridae* and related viruses. Phylogenetic analysis of orf 2 encoding the nonstructural proteins hel, prot, and RdRp. Fifty-six sequences of noraviruses and nora-like viruses were aligned with the help of ClustalW and used for tree inference with IQ-TREE 2.1.3. Optimal substitution model: Q.pfam+F+R6. Numbers at nodes indicate ultrafast bootstrap support obtained with 10,000 replications. Presented is the maximum likelihood tree. It is arbitrarily rooted with the clade of nora-like viruses, which exhibit only one orf and lack nora-VP4-like capsid proteins. The scale bar indicates the number of substitutions per site. Color code: blue, classified reference virus; red, Teltow Canal viruses (TC-PLVs); black, unclassified viruses. Presented are GenBank accession numbers, species names (printed in bold and in italics), virus names, and strain designations/sequence identifiers (in round brackets). Square brackets indicate clades with different gene layouts. A filled dot (●) indicates a Havel virus. ^§^ Only partial genome available.

**Figure 7:**
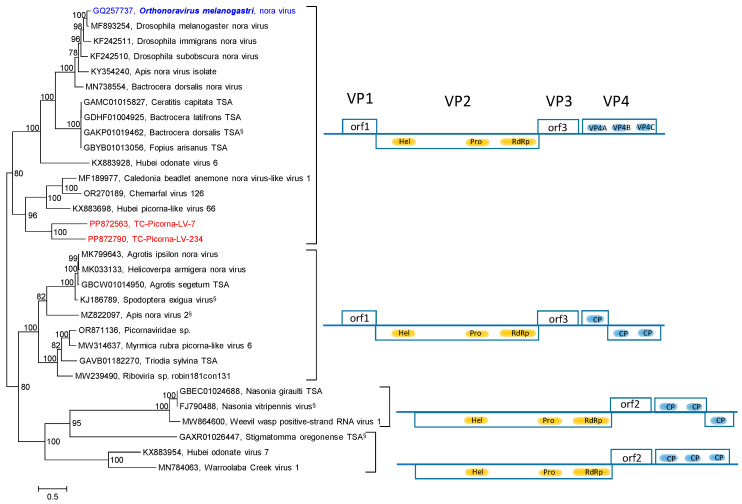
*Noraviridae* and related viruses. Phylogenetic analysis of CP-encoding orfs. Thirty-one sequences of noraviruses and nora-like viruses were aligned with the help of ClustalW and used for tree inference with IQ-TREE 2.1.3. Optimal substitution model: Q.pfam+F+I+G4. Presented is the midpoint-rooted maximum likelihood tree. Numbers at nodes indicate bootstrap support obtained with 1000 replications. The scale bar indicates the number of substitutions per site. Color code: blue, classified reference virus; red, Teltow Canal viruses (TC-PLVs); black, unclassified viruses. Presented are GenBank accession numbers, species names (printed in bold and in italics), and virus names. Square brackets indicate groups of viruses with a similar genome organization. Gene layouts are presented in the right panel, with boxes indicating the orfs. Gene products corresponding to noravirus VP2 products are highlighted in yellow; those equivalent to noravirus VP4 are in blue. The functions of noravirus orf 1 and orf 3 are unknown. Abbreviations: CP, capsid protein; hel, helicase; orf, open reading frame; prot, proteinase; RdRp, RNA-dependent RNA polymerase; VP, viral protein. ^§^ Only partial genome available.

Our RdRp tree included noraviruses and nora-related viruses, eleven sequences altogether, plus four nora-related viruses from the Teltow Canal and one virus from the Havel River (Figure S2). The RdRp tree indicates a monophyletic clade, although the genome layouts of these viruses differ significantly. For a further characterization, the nonstructural polyprotein sequences (matching the noravirus VP2) of 56 viruses were aligned and used to infer another phylogenetic tree (Figure 6). Seven groups correspond to different genome layouts with one to five orfs. Thirty CP sequences with similarity to the noravirus structural polyprotein precursor VP4 were also analyzed (Figure 7). This analysis did not include the nora-like viruses with one and two orfs, as they do not possess VP4-like sequences. The VP4 tree shows four groups, each with a variant genome layout. Both analyses indicate that TC-PLV-7, -234, and -425 are closely related to the noraviruses, whereas TC-PLV-411 has similarity to HPLV-4 and clusters with nora-related viruses with only two orfs.

#### 3.2.8. Posaviruses and Related Viruses

Posaviruses have originally been described as porcine stool-associated viruses [33,34,35,36,37]. Meanwhile, however, posaviruses and posa-like viruses have been detected in samples of pigs, humans, chimpanzees, rats, bats, and carps [9,38,39,40,41,42]. Furthermore, similar viruses have been detected in insects [42], freshwater mussels [11], seaweed [43], pig nematodes [5,44], and environmental samples [8]. Posaviruses and posa-like viruses have monocistronic genomes that encode a polyprotein with nonstructural proteins (helicase, proteinase, and RdRp) located at the N-terminal part and three CPs with a jellyroll fold at the C-terminal part.

**Figure 8:**
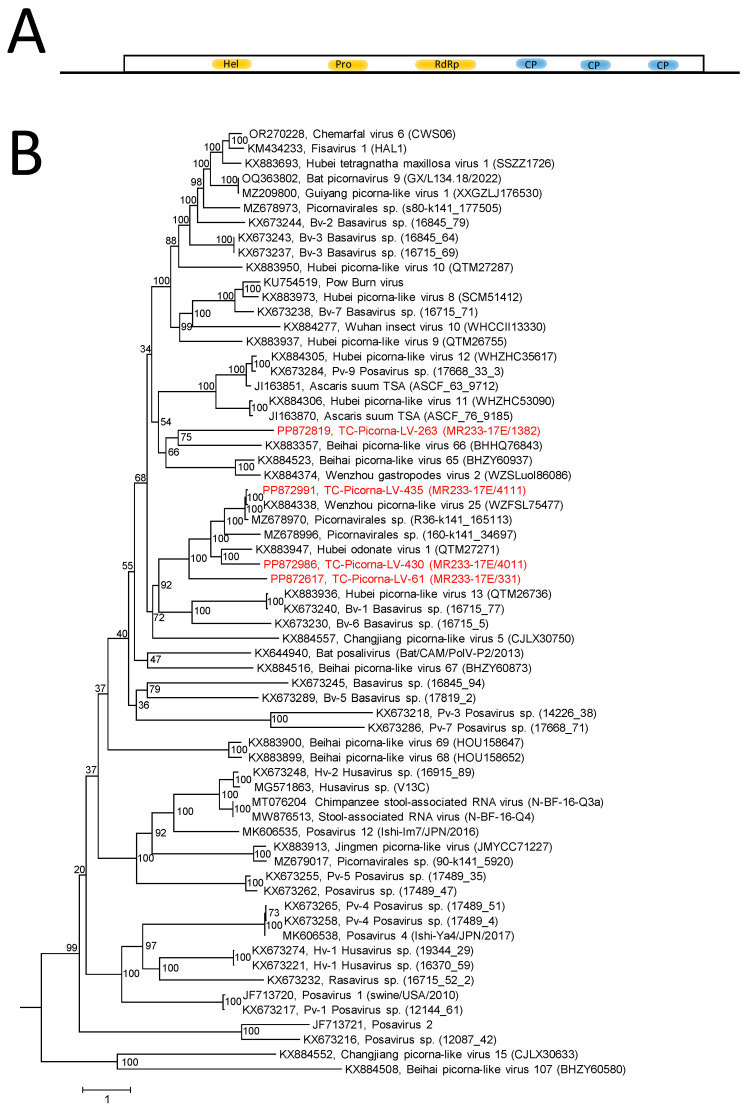
Posaviruses and related viruses. (**A**) Genome organization of posaviruses. The single orf is represented by a box. The array of nonstructural proteins (hel, pro, and RdRp) and CPs with a jellyroll motif is indicated. (**B**) Phylogenetic analysis of hel/pro/RdRp-encoding sequences. Sixty-four sequences of posaviruses and posa-like viruses were aligned with the help of ClustalW and used for tree inference with IQ-TREE 2.1.3. Optimal substitution model: Q.pfam + F + R6. Presented is the maximum likelihood tree. The maximum likelihood tree is rooted with Changjiang picorna-like virus 15 and Beihai picorna-like virus 107 as the outgroups. Numbers at nodes indicate ultrafast bootstrap support obtained with 10,000 replications. The scale bar indicates the number of substitutions per site. Color code: red, Teltow Canal viruses (TC-PLVs); black, unclassified viruses. Presented are GenBank accession numbers, virus names, and strain designations/sequence identifiers (in round brackets).

The RdRp tree (Figure S2) indicates a distinctly marked clade of 23 viruses including 5 TC-PVLs. For further characterization, sequences of the helicase, proteinase, and RdRp domains of 58 posaviruses and posa-like viruses from GenBank, 4 TC-posa-like viruses with suited sequences, plus 2 other viruses with monocistronic genomes (serving as an outgroup) were aligned and used for tree inference (Figure 8). The resulting tree has many long branches suggesting the existence of at least two dozen yet-to-be-defined taxa. TC-PLV-435 is very similar to Wenzhou picorna-like virus 25, and TC-PLV-430 is related to Hubei odonate virus 1. Furthermore, two viruses, TC-PLV-61 and -263, are highly divergent and represent novel posa-like viruses.

## 4. Discussion

We have previously reported the identification of genomic RNA from 166 picorna-like viruses in a 50 L water sample collected in July 2018 from the Havel River in Berlin, Germany [8]. Several findings of that study were unexpected: (i) the presence of a great number of marna-like and dicistro-like viruses; (ii) the detection of only one picornavirus (ampivirus A2), and no evidence of secoviruses; and (iii) the description of viruses with unusual dicistronic gene arrays. In order to substantiate our previous findings, we searched for picorna-like viruses in a water sample that was collected 11 months earlier from the Teltow Canal, another waterway in Berlin, Germany. The Teltow Canal sample was richer in yield: 66,371 virus-specific metaSPAdes scaffolds were identified using DIAMOND versus 16,922 scaffolds from the Havel River sample. Likewise, the clc assembler yielded 41,408 virus-specific contigs from the Teltow Canal sample but only 8810 contigs from the Havel River sample. In the present study, a total of 7386 scaffolds/5587 contigs were assigned using DIAMOND to the *Picornavirales* order, of which 513 genomes were further analyzed, revealing 239 (46.6%) complete and 274 partial genomes (53.4%). Seventy-one TC-PLVs (13.8%) had amino acid sequence similarities greater than 85% to the Havel picorna-like viruses, which were described previously [8], and seventy TC-PLV sequences (13.6%) showed similarities greater than 85% to other virus sequences deposited in the Genbank. Hence, many of our TC-PLV sequences were novel (<85% similarity to known viruses in GenBank). In an attempt to assign our TC-PLVs to the acknowledged *Picornavirales* families, the RdRp sequences were aligned and used to infer a phylogenetic tree (Figure 2 and Figure S2). This tree indicates that 158 TC-PLVs belong to the *Marnaviridae* family. Three viruses are likely cripaviruses of the family *Dicistroviridae* and 95 viruses are candidates of this family. Four chipolyciviruses were identified as well as two viruses of a putatively novel genus of *Polycipiviridae*. Furthermore, three new iflaviruses (family *Iflaviridae*), one Aichi virus (genus *Kobuvirus* of *Picornaviridae*), one ampivirus (genus *Ampivirus* of *Picornaviridae*), and eight new candidate viruses of *Picornaviridae* were detected, as well as eleven solinvi-like viruses and four nora-like viruses. Altogether, 287 TC-PLVs (55.9%) could be assigned to *Picornavirales* families, but 106 viruses (20.7%) escaped from our attempts to assign them to any of the 9 *Picornavirales* families on the basis of their RdRp sequences. In addition, 120 sequences (23.4%) lacked RdRp sequences but showed similarity to the *Picornavirales* hallmarks helicase, proteinase, or CP genes.

The prevalence of marna-like viruses in our Teltow Canal sample contrast with the perception that viruses of *Marnaviridae* are marine RNA viruses. This view is supported by the fact that 8 of the 20 acknowledged *Marnaviridae* species have been isolated from marine organisms (raphidophytes, diatoms, and thraustochytrids). The remaining species were detected using metagenomics in coastal and oceanic marine water samples (https://ictv.global/report/chapter/marnaviridae/marnaviridae, accessed on 12 May 2024, [25]). However, evidence is accumulating that marna-like viruses are also present in freshwater ecosystems or are associated with higher plants and fecal samples [8,34,40,45]. The identification of an additional 158 genome sequences of marna-like viruses in a Teltow Canal water sample supports the hypothesis that protists from freshwater bodies also harbor such viruses. Remarkably, viruses of the *Locarnavirus* genus predominate, members of the genera *Labyrnavirus*, *Marnavirus*, *Salisharnavirus*, and *Sogarnavirus* are less prevalent, and no viruses of the *Bacillarnavirus* and *Kusarnavirus* genera were detected in our sample. Whether this observation is the result of biased sampling, biased virus enrichment, biased RNA extraction, or reflects different prevalences of viruses and host organisms, has to be investigated in future studies. Twenty TC-PLVs and many marna-like viruses from other studies could not be assigned to any of the seven *Marnaviridae* genera on the basis of their RdRp sequence. The creation of additional species and genera appears reasonable to adequately describe the virus diversity of the *Marnaviridae* clade. For example, the PLVs with monocistronic genome organization need consideration. We identified 26 monocistronic TC-PLVs that are scattered on the *Locarnavirus* branch, with exception of one major clade (Figure S2). In addition, one monocistronic strain is marna-like, and four viruses are untypeable.

Dicistro-like viruses constitute the second largest virus group in our sample. However, although 98 TC-PLV sequences clustered on the branch with dicistrovirus reference strains, only 3 sequences could be assigned to *Cripavirus drosophilae* and *Cripavirus mortiferum*, respectively. The remaining 95 sequences have too diverse sequences for proper classification. As with the members of the *Marnaviridae* and the marna-like viruses, dicistroviruses and their many related viruses are also in need of an adequate classification. Noteworthy are four viruses that cluster close to the aparaviruses but have a monocistronic gene layout. Another interesting strain is the dicistronic TC-PLV-57 because of its reverse order of the hel-pro-pol replication block and the CP polyprotein precursor. This virus has a CP-encoding orf at the 5’-part of the genomic RNA and is the first virus with this gene layout that clusters with dicistroviruses.

PLVs are characterized by a conserved set of genes, the viral hallmark genes, comprising gene regions which encode the helicase, proteinase, RdRp, and CPs with jellyroll folds. Their diversification is assumed to have happened concomitantly with the evolution of eukaryogenesis [46]. Presumably, all other PLV gene regions were acquired by gene exchange, but mechanisms and donors have not been identified as of yet. However, dicistronic or multicistronic expression mechanisms employing IRESs, subgenomic RNAs, or -1 frameshift signals have been found in various virus orders and suggest horizontal gene transfer, even with distantly related viruses. The presence of hepatitis C virus IRES in several picornavirus genera [47] and the emergence of porcine enteroviruses with a torovirus proteinase [48,49,50] are more recent examples of possible horizontal gene transfer events. Whether horizontal gene transfer between the nine families of the *Picornavirales* occurs is not known, but the variety of genome layouts suggest repeated gross modifications (compare Figure S1). However, clear proof is lacking, and the available phylogenetic trees give no indications. In contrast, phylogenetic analyses of plant-associated viruses indicate the rampant exchange of capsid protein-encoding genes; for recent examples, see [17].

The *Picornavirales* virome of the Teltow Canal exhibits a striking disproportional prevalence of marna- and dicistroviruses. This imbalance may be explained by the sampling time: our water sample was taken in July 2017, when unicellular algae, diatoms, and other protists, which are supposed to harbor marnaviruses, propagate well at warm ambient temperatures. Likewise, the population density of (aquatic) arthropods, which are hosts of dicistroviruses, is also high in summer.

Among the 226 TC-PLVs that cannot be assigned to existing genera and families are 5 viruses that are posa-like. Posaviruses comprise a well-separated clade with strong bootstrap support in the RdRp tree. They possess a monocistronic gene layout with the nonstructural protein-encoding sequences at the 5′-part of the genomic RNA. This property might justify the creation of another family in the order *Picornavirales*, although the within-clade heterogeneity is high, suggesting the existence of several genera and up to 20 species. The detection of posa-like viruses and Aichi virus indicates that the water of the Teltow Canal is burdened with enteric viruses of humans and domestic animals. We have previously shown that certain plant viruses like pepper mild mottle virus were prevalent in high titers in the same Teltow Canal sample [17]. The detection of enteric viruses is compatible with the presence of pepper mild mottle virus as an indicator of fecal contamination [51]. In the summer months, the Teltow Canal receives the discharge of a municipal wastewater treatment plant to relieve the Havel River, which is intensively used for recreational activities at that time. A time-series investigation of the virome compositions of both rivers seems appropriate to study, in more detail, the various virus prevalences in the course of the year.

## 5. Conclusions

The *Picornavirales* order is a highly diverse order of positive-strand RNA viruses. Despite all variability regarding their genome size, gene layout, and host range, all members are characterized by a conserved helicase, proteinase, polymerase, and one to three capsid proteins with a jellyroll fold. The order presently comprises 9 virus families with 105 genera and 371 species, but more than 2000 unclassified candidate viruses have been described with a predominance of marine PLVs. In order to understand whether this abundance reflects a natural situation or a biased record, we searched our Teltow Canal dataset for sequences of PLVs. As a result, we added 513 sequences of mostly new viruses to the assemblage of unassigned PLVs. About 370 sequences of our dataset exhibit less than 85% amino acid similarity to known picorna-like viruses. Our data demonstrate the presence of many marna-like viruses in riverine freshwater, the predominance of viruses associated with protists and arthropods, and some burden with municipal wastewater. The detection of several viruses with unusual gene layouts underscores the need to continue such investigations in order to conceive the sequence space of picorna-like viruses and to accommodate these viruses in a topical taxonomy.

## Figures and Tables

**Table 1:** Preliminary assignment of TC-PLVs based on phylogenetic analyses.

Family	Genus	No. of Sequences	Σ
*Caliciviridae*	11 genera	0	0
*Dicipiviridae* *	*Aparavirus*	0	98
	*Cripavirus*	3	
	*Triatovirus*	0	
	Untypeable	95	
*Iflaviridae* *	*Iflavirus*	3	3
*Marnaviridae* *	*Bacillarnavirus*	0	158
	*Kusarnavirus*	0	
	*Labyrnavirus*	10	
	*Locarnavirus*	106	
	*Marnavirus*	3	
	*Salisharnavirus*	8	
	*Sogarnavirus*	11	
	Untypeable	20	
*Noraviridae* *	*Orthonoravirus*	0	4
	Untypeable	4	
*Picornaviridae* *	*Ampivirus*	1	10
	*Kobuvirus*	1	
	66 other genera	0	
	Untypeable	8	
*Polycipiviridae* *	*Chipolycivirus*	4	6
	*Hupolycivirus*	0	
	*Sopolycivirus*	0	
	Untypeable	2	
*Secoviridae*	8 genera	0	0
*Solinviviridae* *	*Invictavirus*	0	11
	*Nyfulvavirus*	0	
	Untypeable	11	
Unclassified family	Unclassified genus	106	106

* and related viruses.

## Data Availability

BioProject ID: PRJNA1113981; Biosample: SAMN41472818; Short Read Archive: SRR29096028; GenBank accession numbers: PP872545-PP873057.

## Supplementary Materials

The following supporting information can be downloaded at: https://www.mdpi.com/article/10.3390/v16071020/s1, Figure S1: Schematic genome layouts of picorna-like viruses; Figure S2: Phylogenetic analysis of RdRp sequences; Figure S3: Phylogenetic analysis of picornavirus capsid protein sequences; Table S1: Information on virus sequences.
