# Supplementary material for: Diversity of Picorna-Like Viruses in the Teltow Canal, Berlin, Germany
Source: Viruses. 2024 Jun 25;16(7):1020. doi: 10.3390/v16071020 (PMC11281612; doi:10.3390/v16071020)
Supplement: Supplementary file 1 [file viruses-16-01020-s001.zip › viruses-3072729-supplementary.pdf]

viruses

MDPI

# Diversity of picorna-like viruses of the Teltow Canal, Berlin, Germany

## Supplementary Information

**Roland Zell [1]*, Marco Groth [2], Lukas Selinka [1], and Hans-Christoph Selinka [3,]**

[1] Section of Experimental Virology, Institute for Medical Microbiology, Jena University Hospital, Friedrich Schiller University, Jena, Germany
[2] CF Next Generation Sequencing, Leibniz Institute on Aging, Fritz Lipmann Institute, Jena, Germany
[3] Section II 1.4 Microbiological Risks, Department of Environmental Hygiene, German Environment Agency, Berlin, Germany

**Legends to supplementary figures:**

**Supplementary Figure S1: Schematic genome layouts of picorna-like viruses.** Genomes are indicated by thick lines. Arrows represent open reading frames. Hallmark genes encoding helicase, proteinase and polymerase are lighlighted in yellow, genes encoding structural proteins with jellyroll fold in blue. Each family of the order *Picornavirales* is represented by one to six examples demonstrating the variety of PLV genomes. Abbreviations: DSR, dsRNA-binding protein; FSD, frameshift domain; Hel, helicase; IRES, internal ribosomal entry site; JR, capsid protein with jellyroll fold; orf, open reading frame; OTU, ovarian tumor domain; PLA2, phospholipase A2-like protein; Pro, proteinase; RdRp, RNA-dependent RNA polymerase; sgRNA, subgenomic RNA; VP, viral protein; VPg, viral protein genome-associated.

**Supplementary Figure S2: Phylogenetic analysis of RdRp sequences.** 934 RdRp amino acid sequences representing all nine families of the *Picornavirales* order plus numerous unclassified viruses were aligned with the help of ClustalW. The alignment comprises 111 classified viruses (reference viruses indicated with a #), 398 viruses from the Teltow Canal (TC-PLVs; indicated with a ▲), 100 viruses from the Havel River in Berlin (HPLVs; indicated with a ●), and 330 unclassified virus sequences downloaded from GenBank. The maximum likelihood tree was infered with IQ-TREE 2.1.3 using the Q.pfam+F+R9 substitution model. Ultrafast bootstrap support was obtained with 10,000 replications. Colours indicate acknowledged or assumed membership to one of the nine families of the *Picornavirales* order and the posa-like virus clade. Colour code: purple, *Caliciviridae*; dark blue, *Dicistroviridae*; violet, *Iflaviridae*; dark green, *Marnaviridae*; bright green, *Noraviridae*; magenta, *Picornaviridae*; ochre, *Polycipiviridae*; brown, *Secoviridae*; bright blue, *Solinviviridae*; red, posaviruses and posa-like viruses. Untypeable sequences are printed in black. Presented are GenBank accession numbers, species names (printed in bold face and italics), virus names and strain designations/sequence identifiers (in round brackets). Square brackets indicate clades representing genera of the *Dicipiviridae* and *Marnaviridae* families and subfamilies of the *Picornaviridae*, respectively. The scale bar indicates the number of substitutions per site. Grey boxes highlight viruses with monocistronic genomes, unusual gene layout, and -1 frameshift, respectively.

**Supplementary Figure S3: *Picornaviridae* and related viruses.** Phylogenetic analysis of capsid protein sequences. 164 sequences of picornavirus species, 9 Teltow Canal viruses (TC-PLVs), 1 Havel virus (HPLV) and 6 unclassified picornaviruses were aligned with the help of ClustalW and used for tree inference with IQ-TREE 2.1.3. Optimal substitution model: Q.pfam+F+R7. Numbers at nodes indicate ultrafast bootstrap support obtained with 10,000 replications. The scale bar indicates the number of substitutions per site. Color code: blue, classified reference viruses; red, Teltow Canal viruses; black, unclassified viruses. Presented are GenBank accession numbers, species names (printed in bold and in italics), virus names and strain designations/sequence identifiers (in round brackets). Square brackets indicate the members of the five *Picornaviridae* subfamilies.

Supplementary Figure S1

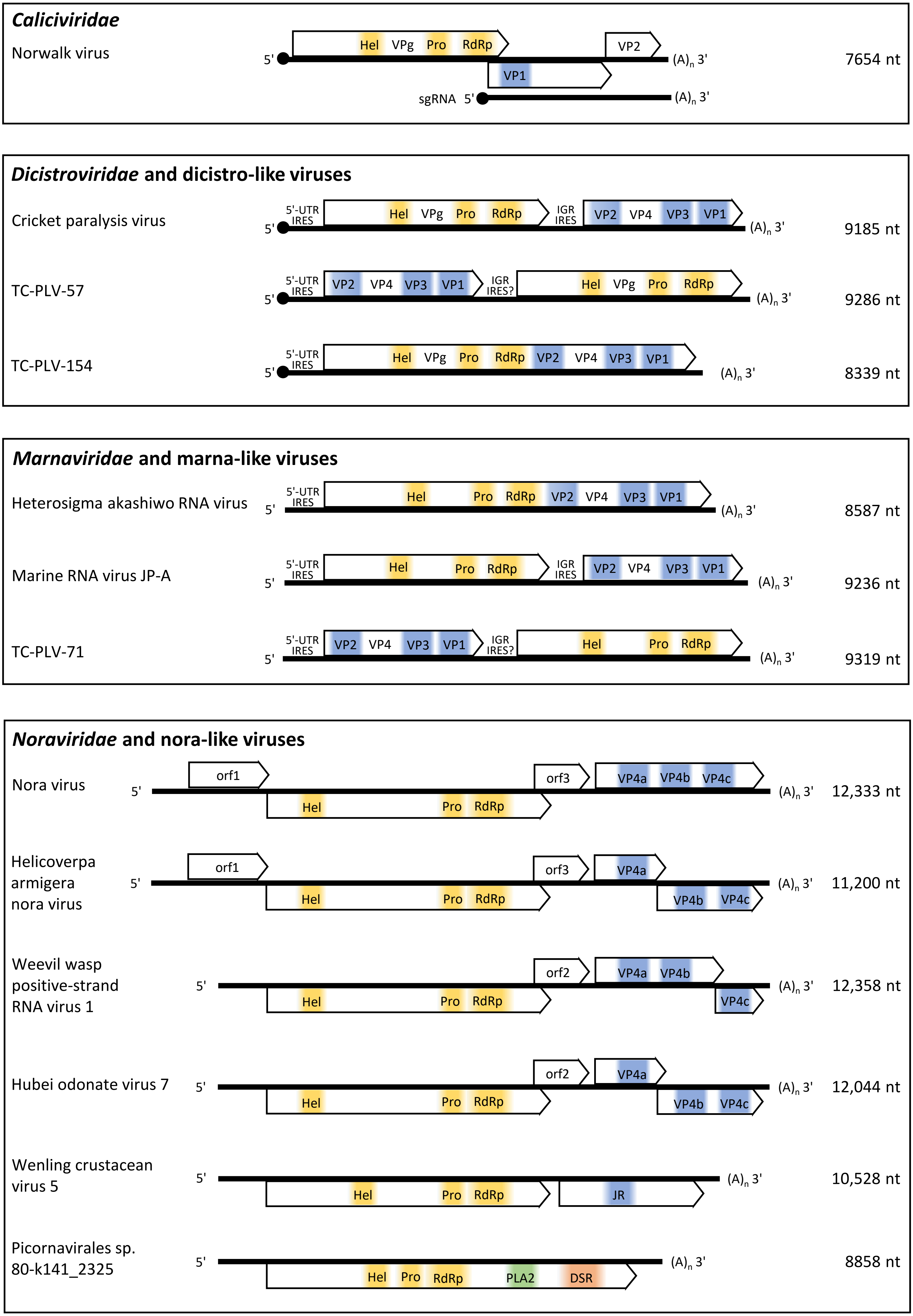

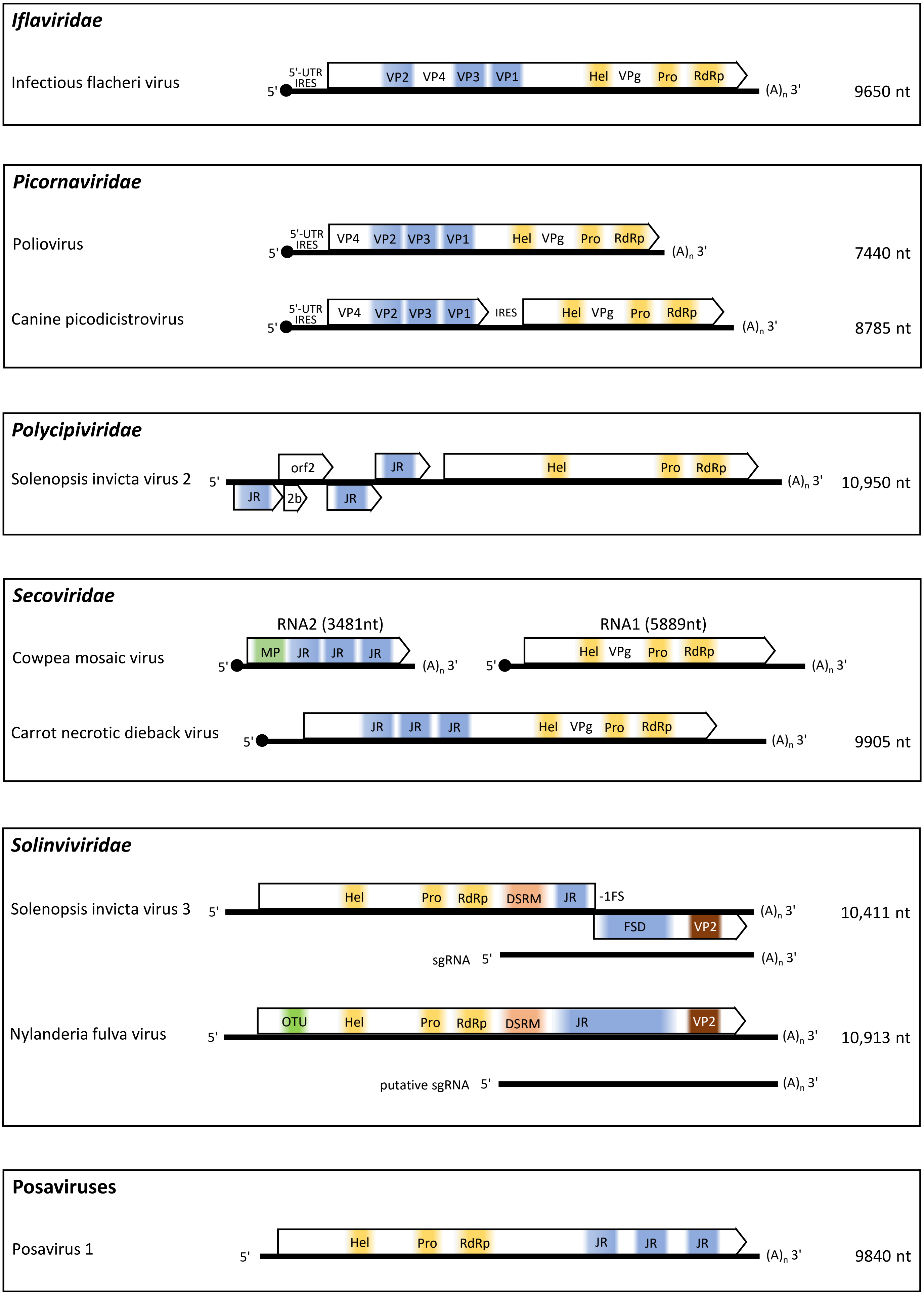
Iflaviridae
Infectious flacheri virus
5'-UTR IRES
5'
VP2 VP4 VP3 VP1 Hel VPg Pro RdRp
(A)n 3'
9650 nt
Picornaviridae
Poliovirus
5'-UTR IRES
5'
VP4 VP2 VP3 VP1 Hel VPg Pro RdRp
(A)n 3'
7440 nt
Canine picodicistrovirus
5'-UTR IRES
5'
VP4 VP2 VP3 VP1 IRES Hel VPg Pro RdRp
(A)n 3'
8785 nt
Polycipiviridae
Solenopsis invicta virus 2
5'
orf2 JR Hel Pro RdRp
JR 2b JR
(A)n 3'
10,950 nt
Secoviridae
RNA2 (3481nt)
RNA1 (5889nt)
Cowpea mosaic virus
5'
MP JR JR JR
(A)n 3'
5'
Hel VPg Pro RdRp
(A)n 3'
Carrot necrotic dieback virus
5'
JR JR JR Hel VPg Pro RdRp
(A)n 3'
9905 nt
Solinviviridae
Solenopsis invicta virus 3
5'
Hel Pro RdRp DSRM JR
-1FS
FSD VP2
(A)n 3'
10,411 nt
sgRNA 5'
(A)n 3'
Nylanderia fulva virus
5'
OTU Hel Pro RdRp DSRM JR VP2
(A)n 3'
10,913 nt
putative sgRNA 5'
(A)n 3'
Posaviruses
Posavirus 1
5'
Hel Pro RdRp JR JR JR
(A)n 3'
9840 nt

Supplementary Figure S2

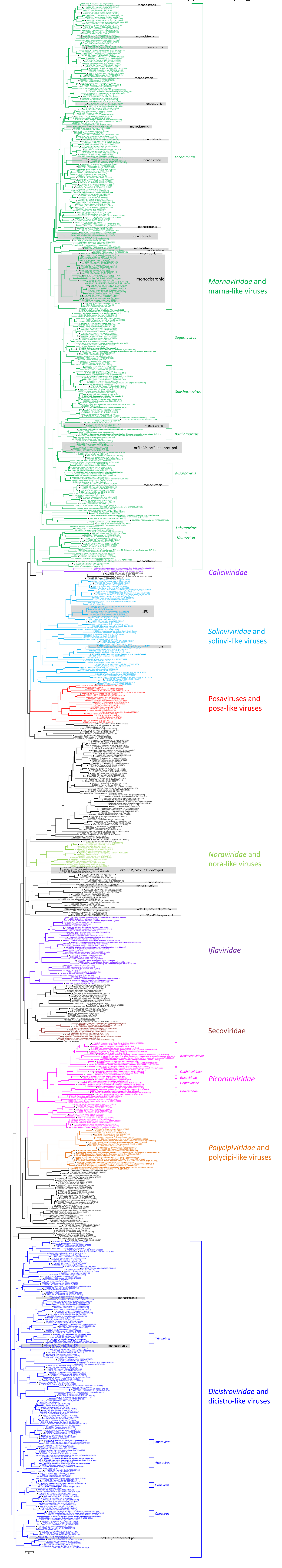

Supplementary Figure S3

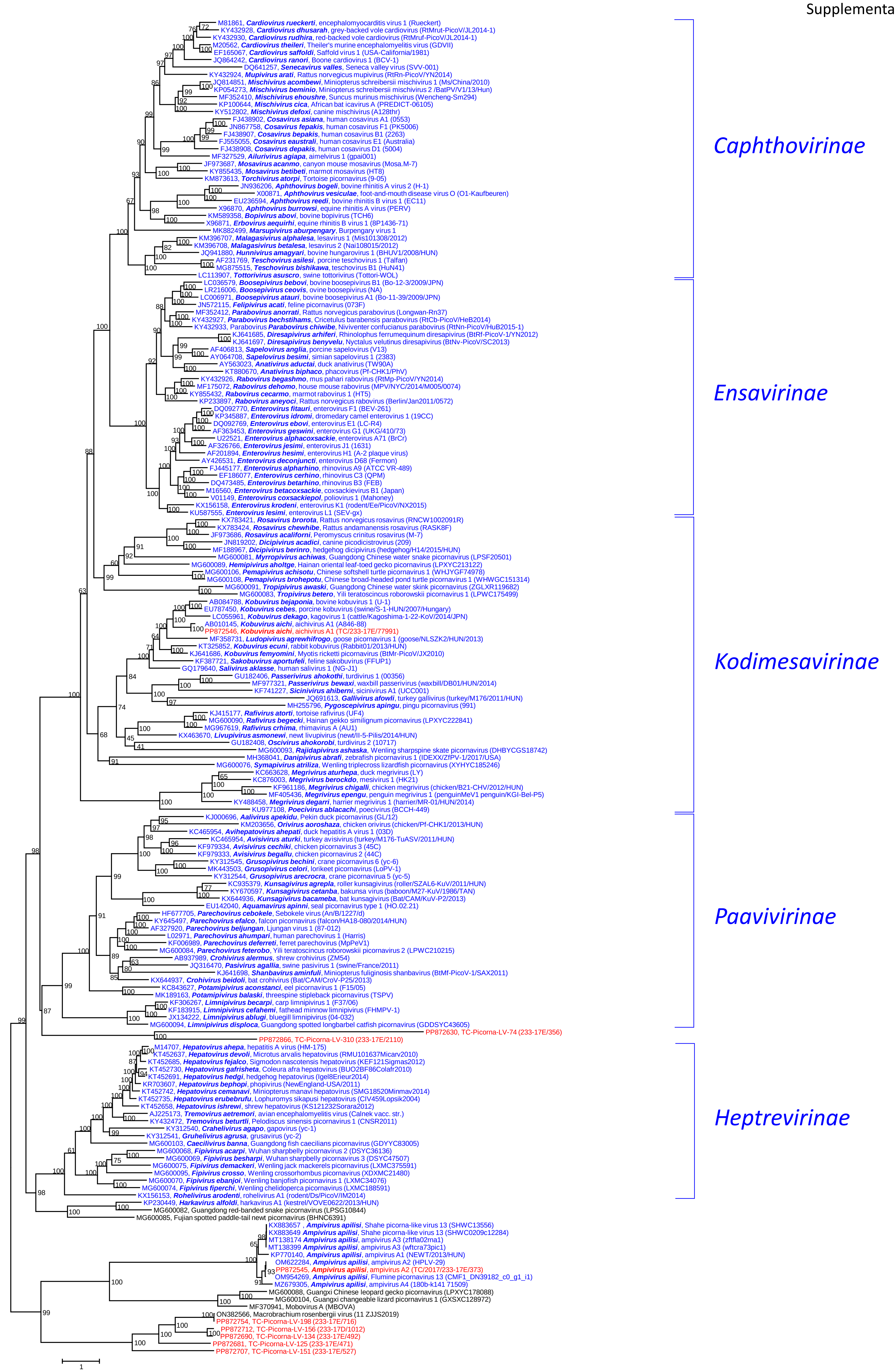

**Table S1**

| GenBank Acc. No. | Virus name | Length | Completeness | Mean depth | Strain designation | Genome layout |
|---|---|---|---|---|---|---|
| PP872545 | **TC-Ampivirus A2** | 9067nt | cg | 269.561 | MR233-17E/373 | monocistr., orf: rhv – rhv – hel – prot – RdRp1 |
| PP872546 | **TC-Aichivirus A1** | 7462nt | par | 7.94296 | MR233-17E/77991 | monocistr., orf-par: rhv – rhv – rhv – prot – hel – prot – RdRp1 |
| PP872547 | **TC-Chipolycivirus 1** | 12123nt | cg | 46.8857 | MR233-17E/118 | orf1: CP; orf2: -; orf3: -; orf4: -; orf5: hel – prot – RdRp1 |
| PP872548 | **TC-Chipolycivirus 2** | 11670nt | cg | 1188.65 | MR233-17E/130 | orf1: CP; orf2: -; orf3: -; orf4: -; orf5: hel – prot – RdRp1 |
| PP872549 | **TC-Chipolycivirus 3** | 11560nt | cg | 598.091 | MR233-17E/135 | orf1: CP; orf2: -; orf3: -; orf4: -; orf5: hel – prot – RdRp1 |
| PP872550 | **TC-Chipolycivirus 4** | 12177nt | cg | 372.734 | MR233-17E/148 | orf1: CP; orf2: -; orf3: -; orf4: -; orf5: DSRM (cd00048, smart00358) – hel – prot – RdRp1 |
| PP872551 | **TC-Cripavirus 1** | 9779nt | cg | 106.147 | MR233-17E/2200 | orf1: hel – prot – RdRp1; orf2: rhv – rhv – CRPV |
| PP872552 | **TC-Cripavirus 2** | 4198nt | par | 16.1475 | MR233-17E/2260 | orf-par: hel – prot – RdRp1 |
| PP872553 | **TC-Cripavirus 3** | 9480nt | cg | 319.783 | MR233-17E/856 | orf1: hel – prot (pfam00548) – RdRp1; orf2: rhv – VP4 – rhv – CRPV |
| PP872554 | **TC-Iflavirus 1** | 9003nt | cg | 36.1731 | MR233-17E/387 | monocistr., orf: rhv – rhv – hel – prot – RdRp1 |
| PP872555 | **TC-Iflavirus 2** | 4874nt | par | 22.7415 | MR233-17E/1907 | orf-par: RING (cd16545, pfam13639) – peptidase C3 (pfam00548) – RdRp1 |
| PP872556 | **TC-Iflavirus 3** | 10022nt | cg | 28.5004 | MR233-17E/227 | orf: rhv – hel – RdRp1 |
| PP872557 | **TC-Picorna-LV-1** | 12961nt | cg | 157.763 | MR233-17E/96 | dicistr., orf1: hel – prot – RdRp1 – rhv; orf2: - |
| PP872558 | **TC-Picorna-LV-2** | 2664nt | par | 10.7425 | MR233-17E/5512 | orf-par: RdRp1 |
| PP872559 | **TC-Picorna-LV-3** | 2703nt | par | 36.9815 | MR233-17E/5374 | orf-par: rhv – VP4 – rhv |
| PP872560 | **TC-Picorna-LV-4** | 4287nt | par | 267.673 | MR233-17D/437 | dicistr., orf1-par: RdRp1; orf2: rhv – VP4 – rhv – CRPV capsid |
| PP872561 | **TC-Picorna-LV-5** | 11511nt | cg | 14994.3 | MR233-18E/137 | monocistr., orf: hel – prot – RdRp1 – rhv – rhv |
| PP872562 | **TC-Picorna-LV-6** | 2699nt | par | 11.2108 | MR233-17E/5390 | orf-par: hel |
| PP872563 | **TC-Picorna-LV-7** | 11182nt | cg | 48.0911 | MR233-17E/151 | 4 orfs, orf1: -; orf2: hel – prot (pfam13365) – RdRp1; orf3: -; orf4: - |
| PP872564 | **TC-Picorna-LV-8** | 10807nt | cg | 33.6651 | MR233-17E/174 | dicistr., orf1: hel – prot – RdRp1; orf2: rhv – rhv |
| PP872565 | **TC-Picorna-LV-9** | 10555nt | cg | 951.906 | MR233-17E/192 | dicistr., orf1: hel – prot (pfam00548) – RdRp1; orf2: rhv – rhv |
| PP872566 | **TC-Picorna-LV-10** | 10251nt | cg | 121.604 | MR233-17E/208 | dicistr., orf1: BIR (cd00022, pfam00653, smart00238) – hel – prot (pfam00548) – RdRp1; orf2: rhv – VP4 – rhv – CRPV |
| PP872567 | **TC-Picorna-LV-11** | 10226nt | cg | 58.6908 | MR233-17E/212 | monocistr., orf: hel – hel – prot – RdRp1 |
| PP872568 | **TC-Picorna-LV-12** | 10104nt | cg | 569.743 | MR233-17E/224 | dicistr., orf1: hel – prot – RdRp1; orf2: rhv – rhv – CRPV |
| PP872569 | **TC-Picorna-LV-13** | 10042nt | cg | 38.4875 | MR233-17E/225 | dicistr., orf1: hel – prot – RdRp1 – TNFRSF4 (cd13406); orf2: VP4 – rhv – rhv |
| PP872570 | **TC-Picorna-LV-14** | 2661nt | cg | 9.96204 | MR233-17E/5521 | dicistr., orf1-par: RdRp1; orf2-par: rhv |
| PP872571 | **TC-Picorna-LV-15** | 9890nt | cg | 70.7319 | MR233-17E/236 | dicistr., orf1: hel – prot – RdRp1; orf2: rhv – rhv |
| PP872572 | **TC-Picorna-LV-16** | 9885nt | cg | 163.215 | MR233-17E/237 | dicistr., orf1: hel – prot – RdRp1; orf2: rhv – rhv |
| PP872573 | **TC-Picorna-LV-17** | 9876nt | cg | 24.7287 | MR233-17E/239 | monocistr., orf: hel – hel – prot – RdRp1 |
| PP872574 | **TC-Picorna-LV-18** | 9831nt | cg | 453.782 | MR233-17E/242 | dicistr., orf1: hel – prot (pfam00548) – RdRp1; orf2: rhv – VP4 – rhv – CRPV |
| PP872575 | **TC-Picorna-LV-19** | 9810nt | cg | 2237.68 | MR233-17E/244 | dicistr., orf1: hel – prot (pfam00548) – RdRp1; orf2: rhv – VP4 – rhv – CRPV |
| PP872576 | **TC-Picorna-LV-20** | 9796nt | cg | 1874.69 | MR233-17E/245 | dicistr., orf1: hel – prot (pfam12381) – RdRp1; orf2: - |
| PP872577 | **TC-Picorna-LV-21** | 9790nt | cg | 75.8046 | MR233-17E/248 | dicistr., orf1: hel – prot – RdRp1; orf2: rhv – VP4 – rhv |
| PP872578 | **TC-Picorna-LV-22** | 9922nt | cg | 87.212 | MR233-17E/250 | dicistr., orf1: hel – prot (pfam00548) – RdRp1; orf2: rhv – VP4 – rhv – CRPV |
| PP872579 | **TC-Picorna-LV-23** | 9775nt | cg | 122.361 | MR233-17E/251 | monocistr., orf: rhv – rhv – hel – prot – RdRp1 |
| PP872580 | **TC-Picorna-LV-24** | 9758nt | cg | 304.092 | MR233-17E/253 | monocistr., orf: hel – prot – RdRp1 – rhv – rhv |
| PP872581 | **TC-Picorna-LV-25** | 9756nt | cg | 32.6357 | MR233-17E/254 | dicistr., orf1: prot – RdRp1; orf2: rhv – VP4 – rhv – CRPV |
| PP872582 | **TC-Picorna-LV-26** | 9697nt | cg | 26.2019 | MR233-17E/257 | dicistr., orf1: hel – prot – RdRp1; orf2: rhv – VP4 – rhv – CRPV |
| PP872583 | **TC-Picorna-LV-27** | 9690nt | cg | 163.694 | MR233-17E/258 | dicistr., orf1: hel – prot – RdRp1; orf2: rhv – rhv |
| PP872584 | **TC-Picorna-LV-28** | 9624nt | cg | 294.525 | MR233-17E/261 | dicistr., orf1: hel – prot – RdRp1; orf2: rhv – rhv – CRPV |
| PP872585 | **TC-Picorna-LV-29** | 9614nt | cg | 236.532 | MR233-17E/262 | monocistr., orf: hel – prot – RdRp1 – DSRM (cd00048, pfam00035, smart00358) |
| PP872586 | **TC-Picorna-LV-30** | 9605nt | cg | 104.952 | MR233-17E/264 | monocistr., orf1: hel – prot – RdRp1 – rhv – rhv – CRPV |
| PP872587 | **TC-Picorna-LV-31** | 9564nt | cg | 2962.96 | MR233-17E/269 | dicistr., orf1: hel – prot – RdRp1; orf2: rhv – rhv |
| PP872588 | **TC-Picorna-LV-32** | 9563nt | cg | 85.9514 | MR233-17E/270 | monocistr., orf: hel – prot – RdRp1 – rhv – rhv – CRPV |
| PP872589 | **TC-Picorna-LV-33** | 9536nt | cg | 5356.25 | MR233-17E/273 | dicistr., orf1: hel – prot – RdRp1; orf2: rhv – VP4 – rhv |
| PP872590 | **TC-Picorna-LV-34** | 9529nt | cg | 990.097 | MR233-17E/274 | monocistr., orf: hel – prot – RdRp1 – rhv – rhv – CRPV |
| PP872591 | **TC-Picorna-LV-35** | 9513nt | cg | 55.6464 | MR233-17E/275 | dicistr., orf1: hel – prot – RdRp1; orf2: SIL1 (pfam16782) – rhv – VP4 -rhv |
| PP872592 | **TC-Picorna-LV-36** | 9489nt | cg | 74.6611 | MR233-17E/277 | dicistr., orf1: DSRM (cd19875) – hel – prot – RdRp1; orf2: rhv – VP4 – rhv – CRPV |
| PP872593 | **TC-Picorna-LV-37** | 9483nt | cg | 46.7686 | MR233-17E/279 | dicistr., orf1: hel – prot – RdRp1; orf2: rhv – rhv |
| PP872594 | **TC-Picorna-LV-38** | 9482nt | cg | 98.8069 | MR233-17E/280 | dicistr., orf1: hel – prot – RdRp1; orf2: rhv – VP4 – rhv – CRPV |
| PP872595 | **TC-Picorna-LV-39** | 9438nt | cg | 355.51 | MR233-17E/286 | monocistr., orf: hel – prot (pfam12381) – RdRp1 – rhv – rhv |
| PP872596 | **TC-Picorna-LV-40** | 9419nt | cg | 79.3707 | MR233-17E/287 | dicistr., orf1: hel – prot – RdRp1; orf2: rhv – rhv |
| PP872597 | **TC-Picorna-LV-41** | 9411nt | cg | 329.356 | MR233-17E/288 | monocistr., orf: hel – prot (pfam12381) – RdRp1 – rhv – rhv |
| PP872598 | **TC-Picorna-LV-42** | 9408nt | cg | 79.6058 | MR233-17E/289 | dicistr., orf1: hel – prot – RdRp1; orf2: rhv – rhv – CRPV |
| PP872599 | **TC-Picorna-LV-43** | 9398nt | cg | 244.809 | MR233-17E/291 | monocistr., orf1: hel – prot – RdRp1 – DSRM (cd00048, pfam00035, smart00358) |
| PP872600 | **TC-Picorna-LV-44** | 9398nt | cg | 48.9251 | MR233-17E/293 | dicistr., orf1: hel – prot – RdRp1; orf2: rhv – rhv – CRPV |
| PP872601 | **TC-Picorna-LV-45** | 9382nt | cg | 24.4709 | MR233-17E/295 | dicistr., orf1: hel – prot – RdRp1; orf2: rhv – VP4 – rhv |
| PP872602 | **TC-Picorna-LV-46** | 9370nt | cg | 16.8126 | MR233-17E/298 | dicistr., orf1: hel – prot – RdRp1; orf2: rhv -VP4 – rhv – CRPV |
| PP872603 | **TC-Picorna-LV-47** | 9369nt | cg | 87.3214 | MR233-17E/299 | dicistr., orf1: hel – prot – RdRp1; orf2: rhv – VP4 – rhv |
| PP872604 | **TC-Picorna-LV-48** | 9369nt | cg | 20.0204 | MR233-17E/300 | dicistr., orf1: hel – prot – RdRp1; orf2: rhv – rhv |
| PP872605 | **TC-Picorna-LV-49** | 9361nt | cg | 24.3076 | MR233-17E/301 | dicistr., orf1: hel – prot – RdRp1; orf2: rhv – rhv |
| PP872606 | **TC-Picorna-LV-50** | 9360nt | cg | 18235.3 | MR233-17E/302 | dicistr., orf1: hel – prot – RdRp1; orf2: rhv – VP4 – rhv – CRPV |

| | | | | | | |
|---|---|---|---|---|---|---|
| PP872607 | **TC-Picorna-LV-51** | 9343nt | cg | 817.911 | MR233-17E/304 | monocistr., orf: hel – prot – RdRp1 – rhv – rhv – CRPV |
| PP872608 | **TC-Picorna-LV-52** | 9340nt | cg | 49.0241 | MR233-17E/305 | dicistr., orf1: hel – prot – RdRp1; orf2: rhv – VP4 – rhv |
| PP872609 | **TC-Picorna-LV-53** | 9333nt | cg | 8779.64 | MR233-17E/307 | dicistr., orf1: hel – prot – RdRp1; orf2: rhv – VP4 – rhv – CRPF-capsid |
| PP872610 | **TC-Picorna-LV-54** | 9313nt | cg | 934.236 | MR233-17E/308 | dicistr., orf1: hel – prot – RdRp1; orf2: rhv – VP4 – rhv |
| PP872611 | **TC-Picorna-LV-55** | 9291nt | cg | 3819.93 | MR233-17E/313 | monocistr., orf: hel – prot – RdRp1 – rhv – rhv |
| PP872612 | **TC-Picorna-LV-56** | 9350nt | cg | 183.527 | MR233-17E/315 | monocistr., orf: hel – prot (pfam12381) – RdRp1 – rhv – rhv – CRPV |
| PP872613 | **TC-Picorna-LV-57** | 9286nt | cg | 22.8908 | MR233-17E/317 | dicistr., orf1: rhv – rhv – CRPV; orf2: hel – prot – RdRp1 |
| PP872614 | **TC-Picorna-LV-58** | 9285nt | cg | 32.0081 | MR233-17E/318 | dicistr., orf1: hel – prot – RdRp1; orf2: rhv – rhv – CRPV |
| PP872615 | **TC-Picorna-LV-59** | 9274nt | cg | 165.332 | MR233-17E/322 | dicistr., orf1: hel – prot – RdRp1; orf2: rhv – VP4 – rhv |
| PP872616 | **TC-Picorna-LV-60** | 9260nt | cg | 11007.1 | MR233-17E/328 | dicistr., orf1: hel – prot – RdRp1; orf2: rhv – VP4 – rhv – CRPV |
| PP872617 | **TC-Picorna-LV-61** | 9239nt | cg | 43.7638 | MR233-17E/331 | monocistr., orf: hel – prot (pfam00548) – RdRp1 – rhv – rhv – CRPV |
| PP872618 | **TC-Picorna-LV-62** | 9237nt | cg | 701.896 | MR233-17E/332 | dicistr., orf1: hel – prot (pfam00548) – RdRp1; orf2: rhv – rhv – CRPV |
| PP872619 | **TC-Picorna-LV-63** | 9235nt | cg | 30.0689 | MR233-17E/333 | dicistr., orf1: hel – prot – RdRp1; orf2: rhv – rhv – CRPV |
| PP872620 | **TC-Picorna-LV-64** | 9415nt | cg | 4083.23 | MR233-17E/334 | monocistr., orf: hel – prot (pfam12381) – RdRp1 – rhv – rhv – CRPV |
| PP872621 | **TC-Picorna-LV-65** | 9229nt | cg | 47.2271 | MR233-17E/337 | dicistr., orf1: hel – prot – RdRp1; orf2: rhv – VP4 – rhv |
| PP872622 | **TC-Picorna-LV-66** | 9214nt | cg | 825.935 | MR233-17E/340 | dicistr., orf1: hel – prot – RdRp1; orf2: rhv – VP4 -rhv |
| PP872623 | **TC-Picorna-LV-67** | 9206nt | cg | 52.379 | MR233-17E/342 | dicistr., orf1: hel – prot – RdRp1; orf2: rhv – rhv – CRPV |
| PP872624 | **TC-Picorna-LV-68** | 9198nt | cg | 228.96 | MR233-17E/345 | monocistr., orf: hel – prot – RdRp1 – rhv – CRPV – rhv |
| PP872625 | **TC-Picorna-LV-69** | 9225nt | cg | 59.7589 | MR233-17E/346 | dicistr., orf1: hel – prot – RdRp1; orf2: rhv – rhv – CRPV |
| PP872626 | **TC-Picorna-LV-70** | 9158nt | cg | 44.3247 | MR233-17E/351 | dicistr., orf1: hel – prot – RdRp1; orf2: rhv – VP4 – CRPV |
| PP872627 | **TC-Picorna-LV-71** | 9319nt | cg | 105.933 | MR233-17E/352 | dicist., orf1: rhv – VP4 -rhv; orf2: hel – prot – RdRp1 |
| PP872628 | **TC-Picorna-LV-72** | 9150nt | cg | 63.2432 | MR233-17E/354 | dicistr., orf1: hel – prot – RdRp1; orf2: rhv – VP4 – rhv |
| PP872629 | **TC-Picorna-LV-73** | 9149nt | cg | 21.6874 | MR233-17E/355 | dicistr., orf1: hel – prot – RdRp1; orf2: rhv – rhv – CRPV |
| PP872630 | **TC-Picorna-LV-74** | 9148nt | cg | 76.8448 | MR233-17E/356 | monocistr., orf: rhv – rhv – hel – prot – RdRp1 |
| PP872631 | **TC-Picorna-LV-75** | 9147nt | cg | 79.2347 | MR233-17E/357 | dicistr., orf1: hel – prot (pfam12381) – RdRp1; orf2: rhv – rhv – CRPV |
| PP872632 | **TC-Picorna-LV-76** | 9145nt | cg | 54.5338 | MR233-17E/358 | moncistr., orf: hel – prot – RdRp1 – rhv – VP4 – rhv |
| PP872633 | **TC-Picorna-LV-77** | 9440nt | cg | 72.2445 | MR233-17E/362 | dicistr., orf1: hel – prot – RdRp1; orf2: rhv – VP4 – rhv |
| PP872634 | **TC-Picorna-LV-78** | 9097nt | cg | 91.9148 | MR233-17E/363 | dicistr., orf1: hel – prot (pfam12381) – RdRp1; orf2: rhv – rhv – CRPV |
| PP872635 | **TC-Picorna-LV-79** | 9094nt | cg | 208.319 | MR233-17E/364 | dicistr., orf1: hel – prot – RdRp1; orf2: rhv – rhv |
| PP872636 | **TC-Picorna-LV-80** | 9089nt | cg | 52.4037 | MR233-17E/367 | dicistr., orf1: hel – prot – RdRp1; orf2: rhv – VP4 – rhv – CRPV |
| PP872637 | **TC-Picorna-LV-81** | 9084nt | cg | 15.319 | MR233-17E/370 | monocistr., orf: hel – prot – RdRp1 – rhv – rhv -CRPV |
| PP872638 | **TC-Picorna-LV-82** | 9071nt | cg | 45.5989 | MR233-17E/372 | dicistr., orf1: hel – prot – RdRp1; orf2: rhv – VP4 – rhv – CRPV |
| PP872639 | **TC-Picorna-LV-83** | 2035nt | cg | 13.4727 | MR233-17E/9186 | dicistr., orf1-par: RdRp1; orf2-par: rhv |
| PP872640 | **TC-Picorna-LV-84** | 9065nt | cg | 304.068 | MR233-17E/376 | dicistr., orf1: hel – prot – RdRp1; orf2: rhv – VP4 – rhv – CRPV |
| PP872641 | **TC-Picorna-LV-85** | 9059nt | cg | 11962.9 | MR233-17E/377 | dicistr., orf1: rhv – rhv – CRPV; orf2: hel – prot – RdRp1 |
| PP872642 | **TC-Picorna-LV-86** | 9051nt | cg | 29.766 | MR233-17E/379 | dicistr., orf1: hel – prot – RdRp1; orf2: rhv – VP4 – rhv |
| PP872643 | **TC-Picorna-LV-87** | 9050nt | cg | 24.844 | MR233-17E/381 | dicistr., orf1: hel – prot – RdRp1; orf2: rhv – rhv |
| PP872644 | **TC-Picorna-LV-88** | 9027nt | cg | 16,0731 | MR233-17E/385 | dicistr., orf1: hel – prot – RdRp1; orf2: rhv – rhv – CRPV |
| PP872645 | **TC-Picorna-LV-89** | 9007nt | cg | 430.55 | MR233-17E/386 | dicistr., orf1: hel – prot (pfam12381) – RdRp1; orf2: rhv – rhv – CRPV |
| PP872646 | **TC-Picorna-LV-90** | 2696nt | par | 11.1131 | MR233-17E/5402 | orf-par: - |
| PP872647 | **TC-Picorna-LV-91** | 9001nt | cg | 209.754 | MR233-17E/388 | dicistr., orf1: hel – prot – RdRp1; orf2: rhv – VP4 – rhv – CRPV |
| PP872648 | **TC-Picorna-LV-92** | 8995nt | cg | 266.849 | MR233-17E/390 | dicistr., orf1: hel – prot – RdRp1; orf2: rhv – VP4 – rhv |
| PP872649 | **TC-Picorna-LV-93** | 8994nt | cg | 94.1961 | MR233-17E/392 | dicistr., orf1: hel – prot – RdRp1; orf2: rhv – VP4 – rhv |
| PP872650 | **TC-Picorna-LV-94** | 8966nt | cg | 578.534 | MR233-17E/394 | dicistr., orf1: hel – prot (pfam12391) – RdRp1; orf2: – |
| PP872651 | **TC-Picorna-LV-95** | 8963nt | cg | 21.871 | MR233-17E/395 | dicistr., orf1: hel – prot – RdRp1; orf2: rhv – rhv – CRPV |
| PP872652 | **TC-Picorna-LV-96** | 8932nt | par | 19.5927 | MR233-17E/399 | dicistr., orf1-par: hel – prot – RdRp1; orf2-par: rhv – rhv |
| PP872653 | **TC-Picorna-LV-97** | 10532nt | cg | 54.9953 | MR233-17E/400 | dicistr., orf1: hel – prot – RdRp1; orf2: rhv – rhv |
| PP872654 | **TC-Picorna-LV-98** | 8923nt | cg | 28.9017 | MR233-17E/403 | dicistr., orf1: hel – prot – RdRp1; orf2: rhv – VP4 – rhv |
| PP872655 | **TC-Picorna-LV-99** | 9044nt | cg | 4770.15 | MR233-17E/404 | monocistr., orf: hel – prot (pfam00548) – RdRp – rhv -rhv |
| PP872656 | **TC-Picorna-LV-100** | 8913nt | cg | 127.649 | MR233-17E/408 | dicistr., orf1: VP4 – rhv -CRPV; orf2: hel – prot (pfam00548) – RdRp1 |
| PP872657 | **TC-Picorna-LV-101** | 8906nt | cg | 90.6466 | MR233-17E/411 | dicistr., orf1: hel – prot – RdRp1; orf2: rhv – VP4 – rhv – CRPV |
| PP872658 | **TC-Picorna-LV-102** | 8904nt | cg | 26.6653 | MR233-17E/412 | dicistr., orf1: hel – prot (pfam00548) – RdRp1; orf2: rhv – rhv – CRPV |
| PP872659 | **TC-Picorna-LV-103** | 8899nt | cg | 35.3457 | MR233-17E/414 | dicistr., orf1: hel – prot – RdRp1; orf2: rhv – VP4 – rhv |
| PP872660 | **TC-Picorna-LV-104** | 8881nt | cg | 16.3267 | MR233-17E/417 | dicistr., orf1: hel – prot (pfam00548/pfam12381) – RdRp1; orf2: rhv – rhv – CRPV |
| PP872661 | **TC-Picorna-LV-105** | 8875nt | cg | 172.11 | MR233-17E/419 | dicistr., orf1: hel – prot (pfam00548) – RdRp1; orf2: rhv – rhv – CRPV |
| PP872662 | **TC-Picorna-LV-106** | 8873nt | cg | 62.8127 | MR233-17E/421 | dicistr., orf1: hel – prot – RdRp1; orf2: rhv – VP4 – rhv |
| PP872663 | **TC-Picorna-LV-107** | 8870nt | cg | 47.7558 | MR233-17E/423 | dicistr., orf1: hel – prot – RdRp1; orf2: rhv |
| PP872664 | **TC-Picorna-LV-108** | 8865nt | cg | 38.9371 | MR233-17E/424 | dicistr., orf1: hel – prot – RdRp1; orf2: rhv – VP4 – rhv |
| PP872665 | **TC-Picorna-LV-109** | 8853nt | cg | 8668.02 | MR233-17E/425 | dicistr., orf1: hel – prot – RdRp1; orf2: rhv – rhv – CRPV |
| PP872666 | **TC-Picorna-LV-110** | 8845nt | cg | 95.3716 | MR233-17E/426 | monocistr., orf: hel – prot – RdRp1 – rhv – VP4 – rhv |
| PP872667 | **TC-Picorna-LV-111** | 9065nt | cg | 58.2683 | MR233-17E/427 | monocistr., orf: hel – prot – RdRp1 – rhv – VP4 – rhv |
| PP872668 | **TC-Picorna-LV-112** | 8822nt | cg | 33.271 | MR233-17E/429 | dicistr., orf1: hel – prot – RdRp1; orf2: rhv – VP4 – rhv |
| PP872669 | **TC-Picorna-LV-113** | 8790nt | cg | 23.1509 | MR233-17E/438 | dicistr., orf1: hel – prot – rdRp1; orf2: rhv – VP4 – rhv |
| PP872670 | **TC-Picorna-LV-114** | 8783nt | cg | 43.3691 | MR233-17E/440 | dicistr., orf1: hel – prot – rdRp1; orf2: rhv – VP4 – rhv |

Table S1

| | | | | | | |
|---|---|---|---|---|---|---|
| PP872671 | **TC-Picorna-LV-115** | 8764nt | cg | 21.489 | MR233-17E/443 | dicistr., orf1: hel – prot – RdRp1; orf2: rhv – VP4 – rhv – CRPV |
| PP872672 | **TC-Picorna-LV-116** | 8753nt | cg | 28.7442 | MR233-17E/445 | dicistr., orf1: hel – prot – RdRp1; orf2: rhv – rhv |
| PP872673 | **TC-Picorna-LV-117** | 8913nt | cg | 73.9283 | MR233-17E/446 | monocistr., orf: hel – prot – RdRp1 – rhv – VP4 – rhv |
| PP872674 | **TC-Picorna-LV-118** | 8731nt | cg | 43.1448 | MR233-17E/447 | dicistr., orf1: hel – prot (pfam12381) – RdRp1; orf2: rhv – rhv – CRPV |
| PP872675 | **TC-Picorna-LV-119** | 8715nt | cg | 93.0559 | MR233-17E/449 | dicistr., orf1: hel – prot – RdRp1; orf2: rhv – VP4 – rhv |
| PP872676 | **TC-Picorna-LV-120** | 8971nt | cg | 37.9152 | MR233-17E/461 | dicistr., orf1: hel – prot – RdRp1; orf2: rhv – rhv – CRPV |
| PP872677 | **TC-Picorna-LV-121** | 8677nt | cg | 63.683 | MR233-17E/462 | dicistr., orf1: hel – prot – RdRp1; orf2: rhv – rhv |
| PP872678 | **TC-Picorna-LV-122** | 8672nt | cg | 32.2741 | MR233-17E/464 | dicistr., orf1: hel – prot (pfam00548) – RdRp1; orf2: rhv – rhv |
| PP872679 | **TC-Picorna-LV-123** | 8648nt | cg | 88.938 | MR233-17E/467 | dicistr., orf1: hel – prot – RdRp1 (pfam23194); orf2: rhv – rhv |
| PP872680 | **TC-Picorna-LV-124** | 8633nt | cg | 2140.32 | MR233-17E/469 | dicistr., orf1: hel – prot – RdRp1 (pfam23169); orf2: rhv – rhv |
| PP872681 | **TC-Picorna-LV-125** | 8604nt | cg | 222.098 | MR233-17E/471 | monocistr., orf: rhv – rhv – hel – prot – RdRp1 |
| PP872682 | **TC-Picorna-LV-126** | 8596nt | cg | 46.2996 | MR233-17E/474 | dicistr., orf1: hel – prot – RdRp1; orf2: rhv – VP4 – rhv |
| PP872683 | **TC-Picorna-LV-127** | 8595nt | cg | 22.1411 | MR233-17E/475 | dicistr., orf1: hel – prot – RdRp1; orf2: rhv – rhv – CRPV |
| PP872684 | **TC-Picorna-LV-128** | 8828nt | cg | 46.897 | MR233-17E/477 | dicistr., orf1: hel – prot – RdRp1; orf2: rhv – VP4 – rhv |
| PP872685 | **TC-Picorna-LV-129** | 8576nt | cg | 66.1678 | MR233-17E/480 | dicistr., orf1: hel – prot – RdRp1; orf2: rhv – VP4 – rhv |
| PP872686 | **TC-Picorna-LV-130** | 8570nt | cg | 25.3657 | MR233-17E/483 | monocistr., orf-par: hel – prot – RdRp1 – rhv |
| PP872687 | **TC-Picorna-LV-131** | 8569nt | cg | 25.6322 | MR233-17E/484 | monocistr., orf: hel – prot – RdRp1 – rhv – rhv – CRPV |
| PP872688 | **TC-Picorna-LV-132** | 8554nt | cg | 42.0573 | MR233-17E/487 | dicistr., orf1: hel – prot – RdRp1; orf2: rhv – VP4 – rhv – CRPV |
| PP872689 | **TC-Picorna-LV-133** | 8538nt | cg | 293.058 | MR233-17E/491 | dicistr., orf1: hel – prot – RdRp1; orf2: rhv – rhv – CRPV |
| PP872690 | **TC-Picorna-LV-134** | 8538nt | cg | 30.2456 | MR233-17E/492 | monocistr., orf: rhv – rhv – hel – prot – RdRp1 |
| PP872691 | **TC-Picorna-LV-135** | 8533nt | cg | 26.3156 | MR233-17E/493 | dicistr., orf1: hel – prot – RdRp1; orf2: rhv – VP4 – rhv |
| PP872692 | **TC-Picorna-LV-136** | 8530nt | cg | 211.578 | MR233-17E/494 | monocistr., orf: hel – prot – RdRp1 – Calici-coat (pfam00915) |
| PP872693 | **TC-Picorna-LV-137** | 8527nt | cg | 48.8146 | MR233-17E/495 | monocistr., orf: hel – prot (pfam12381) – RdRp1 – rhv – rhv – CRPV |
| PP872694 | **TC-Picorna-LV-138** | 8506nt | cg | 27.54 | MR233-17E/498 | dicistr., orf1: hel – prot (pfam00548) – RdRp1; orf2: rhv – VP4 – rhv – CRPV |
| PP872695 | **TC-Picorna-LV-139** | 8504nt | cg | 53.9547 | MR233-17E/499 | dicistr., orf1: hel – prot – RdRp1; orf2: rhv – VP4 – rhv |
| PP872696 | **TC-Picorna-LV-140** | 8502nt | cg | 40.9871 | MR233-17E/500 | monocistr., orf: hel – prot – RdRp1 – rhv – VP4 – rhv |
| PP872697 | **TC-Picorna-LV-141** | 8495nt | cg | 32.5198 | MR233-17E/501 | 3 orfs, orf1: hel – prot -RdRp1; orf2: rhv; orf3: - |
| PP872698 | **TC-Picorna-LV-142** | 8457nt | cg | 19.2329 | MR233-17E/509 | dicistr., orf1: hel – prot (pfam00548) – RdRp1; orf2-par: rhv – rhv |
| PP872699 | **TC-Picorna-LV-143** | 8444nt | cg | 46.6248 | MR233-17E/511 | monocistr., orf: hel – prot – RdRp1 – rhv – VP4 – rhv |
| PP872700 | **TC-Picorna-LV-144** | 8438nt | cg | 181.37 | MR233-17E/512 | dicistr., orf1: hel – prot – RdRp1; orf2: rhv – VP4 – rhv |
| PP872701 | **TC-Picorna-LV-145** | 8413nt | cg | 38.8568 | MR233-17E/514 | dicistr., orf1: hel – prot – RdRp1; orf2: rhv – rhv |
| PP872702 | **TC-Picorna-LV-146** | 8409nt | cg | 25.4584 | MR233-17E/517 | dicistr., orf1: hel – prot – RdRp1; orf2: rhv – rhv |
| PP872703 | **TC-Picorna-LV-147** | 8398nt | cg | 38.6173 | MR233-17E/519 | dicistr., orf1: hel – prot – RdRp1; orf2: rhv – VP4 – rhv – CRPV |
| PP872704 | **TC-Picorna-LV-148** | 8380nt | cg | 261.727 | MR233-17E/522 | monocistr., orf: (hel?) – prot – RdRp1 – rhv – VP4 – rhv |
| PP872705 | **TC-Picorna-LV-149** | 8343nt | cg | 20.858 | MR233-17E/525 | dicistr., orf1: hel – prot – RdRp1 , orf2: rhv – VP4 – rhv – CRPV |
| PP872706 | **TC-Picorna-LV-150** | 8356nt | cg | 157.258 | MR233-17E/526 | dicistr., orf1: hel – prot (pfam00548) – RdRp1; orf2: rhv – rhv |
| PP872707 | **TC-Picorna-LV-151** | 8354nt | cg | 26.6471 | MR233-17E/527 | monocistr., orf: rhv – rhv – hel – prot – RdRp1 |
| PP872708 | **TC-Picorna-LV-152** | 8345nt | cg | 37.6845 | MR233-17E/529 | dicistr., orf1: hel – prot – RdRp1; orf2: rhv |
| PP872709 | **TC-Picorna-LV-153** | 8340nt | cg | 7010.83 | MR233-17E/531 | dicistr., orf1: hel – prot – RdRp1; orf2: rhv – rhv |
| PP872710 | **TC-Picorna-LV-154** | 8339nt | cg | 240.369 | MR233-17E/532 | monocistr., orf: hel – prot – RdRp1 – rhv – rhv – CRPV |
| PP872711 | **TC-Picorna-LV-155** | 8303nt | cg | 77.6166 | MR233-17E/538 | dicistr., orf1: hel – prot – RdRp1; orf2: rhv – VP4 – rhv – CRPV |
| PP872712 | **TC-Picorna-LV-156** | 8651nt | cg | 172.658 | MR233-17E/540 | monocistr., orf: rhv – rhv – hel – prot – RdRp1 |
| PP872713 | **TC-Picorna-LV-157** | 8193nt | cg | 234.189 | MR233-17E/541 | dicistr., orf1: hel – prot (pfam00548) – RdRp1; orf2: rhv – VP4 – rhv – CRPV |
| PP872714 | **TC-Picorna-LV-158** | 8288nt | par | 14.8628 | MR233-17E/543 | dicistr., orf1-par: hel – prot – RdRp1; orf2: rhv – rhv |
| PP872715 | **TC-Picorna-LV-159** | 8284nt | cg | 1909.65 | MR233-17E/544 | dicistr., orf1: hel – prot (pfam00548) – RdRp1; orf2: rhv – rhv |
| PP872716 | **TC-Picorna-LV-160** | 8277nt | cg | 95.1917 | MR233-17E/546 | monocistr., orf: hel – prot – RdRp1 – rhv – rhv |
| PP872717 | **TC-Picorna-LV-161** | 8546nt | cg | 20.1755 | MR233-17E/547 | dicistr., orf1: hel – prot – RdRp1; orf2: rhv – VP4 – rhv |
| PP872718 | **TC-Picorna-LV-162** | 8241nt | cg | 111.223 | MR233-17E/551 | dicistr., orf1: hel – prot (pfam00548) – RdRp1; orf2: rhv – rhv – CRPV |
| PP872719 | **TC-Picorna-LV-163** | 8226nt | cg | 44.0791 | MR233-17E/553 | dicistr., orf1: hel – prot – RdRp1; orf2: rhv – VP4 – rhv |
| PP872720 | **TC-Picorna-LV-164** | 8167nt | cg | 114.49 | MR233-17E/558 | dicistr., orf1: hel – prot (pfam00548) – RdRp1; orf2: rhv – rhv |
| PP872721 | **TC-Picorna-LV-165** | 8153nt | cg | 32.7703 | MR233-17E/561 | dicistr., orf1: hel – prot – RdRp1; orf2: rhv – rhv – CRPV |
| PP872722 | **TC-Picorna-LV-166** | 8150nt | cg | 23.5086 | MR233-17E/562 | monocistr., orf: hel – prot (pfam12381) – RdRp1 – rhv – rhv – CRPV |
| PP872723 | **TC-Picorna-LV-167** | 8999nt | cg | 485.683 | MR233-17E/572 | monocistr., orf: hel – prot (pfam12381) – RdRp1 – rhv – rhv – CRPV |
| PP872724 | **TC-Picorna-LV-168** | 8070nt | cg | 22.0751 | MR233-17E/579 | dicistr., orf1: hel – prot – RdRp1; orf2: rhv – rhv |
| PP872725 | **TC-Picorna-LV-169** | 8056nt | cg | 48375.8 | MR233-17E/583 | dicistr., orf1: hel – prot – RdRp1; orf2: rhv – rhv |
| PP872726 | **TC-Picorna-LV-170** | 8038nt | par | 14.2386 | MR233-17E/588 | dicistr., orf1-par: hel – prot – RdRp; orf2: rhv – VP4 – rhv – CRPV |
| PP872727 | **TC-Picorna-LV-171** | 8033nt | cg | 10668.6 | MR233-17E/589 | dicistr., orf1: hel – prot – RdRp; orf2: rhv – rhv |
| PP872728 | **TC-Picorna-LV-172** | 8029nt | cg | 4304.59 | MR233-17E/590 | dicistr., orf1: hel – prot – RdRp1; orf2: rhv – rhv |
| PP872729 | **TC-Picorna-LV-173** | 8027nt | cg | 2992.71 | MR233-17E/592 | dicistr., orf1: hel – prot (pfam00548) – RdRp1; orf2: rhv – rhv |
| PP872730 | **TC-Picorna-LV-174** | 8024nt | cg | 27.5846 | MR233-17E/596 | dicistr., orf1: hel – prot (pfam00548) – RdRp1 (cd23194; orf2: rhv – rhv – CRPV |
| PP872731 | **TC-Picorna-LV-175** | 8513nt | cg | 92.2008 | MR233-17E/599 | dicistr., orf1: rhv – rhv – CRPV; orf2: hel – prot – RdRp1 |
| PP872732 | **TC-Picorna-LV-176** | 8002nt | cg | 5669.6 | MR233-17E/600 | dicistr., orf1: hel – prot (pfam00548) – RdRp1; orf2: rhv – rhv |
| PP872733 | **TC-Picorna-LV-177** | 7998nt | cg | 11820.7 | MR233-17E/601 | dicistr., orf1: hel – prot (pfam00548) – RdRp1; orf2: rhv – rhv |
| PP872734 | **TC-Picorna-LV-178** | 7947nt | cg | 637.273 | MR233-17E/607 | dicistr., orf1: hel – prot (pfam00548) – RdRp1; orf2: rhv – rhv |

| | | | | | | |
|---|---|---|---|---|---|---|
| PP872735 | **TC-Picorna-LV-179** | 7935nt | cg | 23.2606 | MR233-17E/610 | monocistr., orf: hel – prot (pfam00548) – RdRp1 – rhv – rhv – CRPV |
| PP872736 | **TC-Picorna-LV-180** | 7919nt | par | 18.4007 | MR233-17E/614 | monocistr., orf-par: hel – prot – RdRp1 – rhv – rhv |
| PP872737 | **TC-Picorna-LV-181** | 7916nt | cg | 24945.1 | MR233-17E/615 | dicistr., orf1: hel – prot – RdRp1; orf2: rhv – rhv |
| PP872738 | **TC-Picorna-LV-182** | 7916nt | cg | 1209.09 | MR233-17E/616 | dicistr., orf1: hel – prot – RdRp1; orf2: rhv – rhv |
| PP872739 | **TC-Picorna-LV-183** | 7890nt | cg | 1266.17 | MR233-17E/620 | dicistr., orf1: hel – prot (pfam00548) – RdRp1; orf2: rhv – rhv |
| PP872740 | **TC-Picorna-LV-184** | 9243nt | cg | 21.1372 | MR233-17E/631 | monocistr., orf: hel – prot (pfam00548) – RdRp1 – rhv – VP4 – rhv – CRPV |
| PP872741 | **TC-Picorna-LV-185** | 8632nt | cg | 3349.33 | MR233-17E/652 | monocistr., orf: hel – prot – RdRp1 – rhv – VP4 – rhv |
| PP872742 | **TC-Picorna-LV-186** | 7696nt | par | 22.3538 | MR233-17E/654 | dicistr., orf1: hel – prot (pfam00548) – RdRp1; orf2-par: rhv – rhv |
| PP872743 | **TC-Picorna-LV-187** | 8297nt | cg | 38.0133 | MR233-17E/656 | monocistr., orf: hel – prot – RdRp1 – rhv – VP4 – rhv |
| PP872744 | **TC-Picorna-LV-188** | 7685nt | cg | 68.342 | MR233-17E/657 | dicistr., orf1: hel – prot – RdRp1; orf2: rhv – VP4 – rhv – CRPV |
| PP872745 | **TC-Picorna-LV-189** | 7673nt | cg | 41.3205 | MR233-17E/661 | dicistr., orf1: hel – prot – RdRp1; orf2: rhv – rhv – CRPV |
| PP872746 | **TC-Picorna-LV-190** | 7629nt | cg | 28.6859 | MR233-17E/667 | dicistr., orf1: hel – prot – RdRp1; orf2: rhv – VP4 – rhv – CRPV |
| PP872747 | **TC-Picorna-LV-191** | 7421nt | par | 16.3645 | MR233-17E/701 | dicistr., orf1: hel – prot – RdRp1; orf2-par: rhv – VP4 |
| PP872748 | **TC-Picorna-LV-192** | 7403nt | cg | 4437.36 | MR233-17E/703 | dicistr., orf1: rhv; orf2: hel – prot (pfam00548) – RdRp1 |
| PP872749 | **TC-Picorna-LV-193** | 7388nt | par | 16.6146 | MR233-17E/707 | monocistr., orf-par: hel – prot – RdRp1 – rhv – rhv – CRPV |
| PP872750 | **TC-Picorna-LV-194** | 7361nt | cg | 432.615 | MR233-17E/711 | dicistr., orf1: rhv – rhv; orf2: hel – prot (pfam00548) – RdRp1 |
| PP872751 | **TC-Picorna-LV-195** | 10792nt | cg | 215.025 | MR233-17E/712 | dicistr., orf1: hel – prot – RdRp1; orf2: rhv – rhv |
| PP872752 | **TC-Picorna-LV-196** | 8811nt | cg | 29.0524 | MR233-17E/713 | dicistr., orf1: hel – prot – RdRp1; orf2: rhv – VP4 – rhv |
| PP872753 | **TC-Picorna-LV-197** | 7355nt | cg | 13.33 | MR233-17E/714 | dicistr., orf1: hel – prot – RdRp1; orf2: rhv – VP4 – rhv |
| PP872754 | **TC-Picorna-LV-198** | 7340nt | par | 27.4493 | MR233-17E/716 | monocistr., orf-par: rhv – rhv – hel – prot – RdRp1 |
| PP872755 | **TC-Picorna-LV-199** | 7277nt | cg | 4173.42 | MR233-17E/723 | dicistr., orf1: rhv; orf2: hel – prot (pfam00548) – RdRp1 |
| PP872756 | **TC-Picorna-LV-200** | 7277nt | cg | 21.6486 | MR233-17E/725 | dicistr., orf1: hel – prot – RdRp1; orf2: rhv – CRPV |
| PP872757 | **TC-Picorna-LV-201** | 7509nt | cg | 63.8676 | MR233-17E/730 | monocistr., orf: hel – prot – RdRp1 – rhv – rhv – CRPV |
| PP872758 | **TC-Picorna-LV-202** | 8525nt | cg | 38.1087 | MR233-17E/731 | dicistr., orf1: hel – prot – RdRp1; orf2: rhv – VP4 – rhv – CRPV |
| PP872759 | **TC-Picorna-LV-203** | 7137nt | cg | 13.893 | MR233-17E/753 | dicistr., orf1: hel – prot – RdRp1; orf2: rhv – rhv |
| PP872760 | **TC-Picorna-LV-204** | 7115nt | cg | 35.2661 | MR233-17E/764 | monocistr., orf: rhv – rhv – rhv – hel – prot – RdRp1 |
| PP872761 | **TC-Picorna-LV-205** | 7094nt | par | 25.6146 | MR233-17E/768 | dicistr., orf1-par: hel – prot – RdRp1; orf2: rhv – rhv |
| PP872762 | **TC-Picorna-LV-206** | 8927nt | cg | 25.7916 | MR233-17E/769 | dicistr., orf1: hel – prot (pfam00548) – RdRp1; orf2: rhv – VP4 – rhv – CRPV |
| PP872763 | **TC-Picorna-LV-207** | 9597nt | cg | 149.499 | MR233-17E/783 | dicistr., orf1: hel – prot – RdRp1; orf2: rhv – VP4 – rhv – CRPV-cpsid |
| PP872764 | **TC-Picorna-LV-208** | 9152nt | cg | 25.1133 | MR233-17E/797 | dicistr., orf1: hel – prot – RdRp1; orf2: rhv – VP4 – rhv |
| PP872765 | **TC-Picorna-LV-209** | 6925nt | par | 30.2566 | MR233-17E/802 | dicistr., orf1: hel – prot – RdRp1; orf2-par: rhv – VP4 |
| PP872766 | **TC-Picorna-LV-210** | 6912nt | par | 41.8056 | MR233-17E/805 | orf-par: hel – prot – RdRp1 |
| PP872767 | **TC-Picorna-LV-211** | 6851nt | par | 26.3081 | MR233-17E/820 | dicistr., orf1: hel – prot – RdRp1; orf2-par: rhv |
| PP872768 | **TC-Picorna-LV-212** | 6849nt | par | 19.9559 | MR233-17E/821 | monocistr., orf-par: hel – prot – RdRp1 – rhv – CRPV – rhv |
| PP872769 | **TC-Picorna-LV-213** | 6823nt | par | 21.3021 | MR233-17E/828 | dicistr., orf1-par: hel – prot – RdRp1; orf2-par: rhv – VP4 – rhv – CRPV |
| PP872770 | **TC-Picorna-LV-214** | 6817nt | par | 14.3421 | MR233-17E/831 | dicistr., orf1-par: hel – prot – RdRp1; orf2: rhv – VP4 – rhv |
| PP872771 | **TC-Picorna-LV-215** | 7182nt | par | 91.2192 | MR233-17E/839 | dicistr., orf1: hel – prot – RdRp1; orf2-par: rhv – VP4 – rhv |
| PP872772 | **TC-Picorna-LV-216** | 7808nt | cg | 1268.83 | MR233-17E/841 | dicistr., orf1: hel – prot – RdRp1; orf2: rhv – rhv |
| PP872773 | **TC-Picorna-LV-217** | 7754nt | cg | 49.4043 | MR233-17E/854 | dicistr., orf1: hel – prot – RdRp1; orf2: rhv – VP4 – rhv – CRPV |
| PP872774 | **TC-Picorna-LV-218** | 6743nt | par | 13.7053 | MR233-17E/855 | dicistr., orf1-par: hel – prot (pfam12381) – RdRp1; orf2: rhv – VP4 -rhv – CRPV |
| PP872775 | **TC-Picorna-LV-219** | 2657nt | par | 20.1012 | MR233-17E/5536 | orf-par: rhv – rhv |
| PP872776 | **TC-Picorna-LV-220** | 6635nt | par | 11.9851 | MR233-17E/885 | dicistr., orf1-par: prot – RdRp1; orf2: rhv |
| PP872777 | **TC-Picorna-LV-221** | 8685nt | cg | 20.3119 | MR233-17E/896 | monocistr., orf: hel – prot – RdRp1 – rhv – VP4 – rhv |
| PP872778 | **TC-Picorna-LV-222** | 6531nt | par | 20.6096 | MR233-17E/912 | dicistr., orf1: hel – prot (pfam00548) – RdRp; orf2-par: rhv |
| PP872779 | **TC-Picorna-LV-223** | 6452nt | par | 30.6744 | MR233-17E/934 | dicistr., orf1-par: prot – RdRp1; orf2: rhv – VP4 – rhv |
| PP872780 | **TC-Picorna-LV-224** | 9220nt | cg | 1027.75 | MR233-17E/939 | dicistr., orf1: hel – prot – RdRp1; orf2: rhv – (VP4?) – rhv – CRPV |
| PP872781 | **TC-Picorna-LV-225** | 6367nt | par | 32.3259 | MR233-17E/951 | dicistr., orf1-par: prot – RdRp1; orf2: rhv – VP4 – rhv – CRPV |
| PP872782 | **TC-Picorna-LV-226** | 6297nt | par | 155.132 | MR233-17E/980 | monocistr., orf-par: hel – prot (pfam12381) – RdRp1 |
| PP872783 | **TC-Picorna-LV-227** | 6249nt | par | 29.1768 | MR233-17E/1004 | dicistr., orf1-par: hel – prot – RdRp1; orf2-par: rhv – rhv – CRPV |
| PP872784 | **TC-Picorna-LV-228** | 6156nt | par | 23.3361 | MR233-17E/1032 | dicistr., orf1-par: prot – RdRp1; orf2: rhv |
| PP872785 | **TC-Picorna-LV-229** | 8861nt | cg | 1170.69 | MR233-17E/1070 | dicistr., orf1: hel – prot – RdRp1; orf2: rhv – rhv – CRPV |
| PP872786 | **TC-Picorna-LV-230** | 6032nt | par | 12.1369 | MR233-17E/1090 | dicistr., orf1-par: hel – prot (pfam00548) – RdRp1; orf2-par: rhv – VP4 – rhv |
| PP872787 | **TC-Picorna-LV-231** | 8597nt | par | 135.273 | MR233-17E/1092 | dicistr., orf1-par: hel – prot (pfam00548) – RdRp1; orf2: rhv – rhv – CRPV |
| PP872788 | **TC-Picorna-LV-232** | 8919nt | cg | 241.36 | MR233-17E/1096 | monocistr., orf: hel – prot – RdRp1 – rhv – VP4 – rhv |
| PP872789 | **TC-Picorna-LV-233** | 6013nt | par | 27.6453 | MR233-17E/1097 | orf-par: hel – prot (pfam00548) – RdRp1 |
| PP872790 | **TC-Picorna-LV-234** | 6009nt | par | 20.3285 | MR233-17E/1101 | 3 orfs: prot – RdRp1 (cd23200); orf2: -; orf3-par: - |
| PP872791 | **TC-Picorna-LV-235** | 6137nt | par | 131.634 | MR233-17E/1109 | orf-par: (hel) – prot – RdRp1 |
| PP872792 | **TC-Picorna-LV-236** | 6856nt | par | 19.7916 | MR233-17E/1111 | dicistr., orf1-par: hel – prot – RdRp1; orf2: rhv – VP4 – rhv – CRPV |
| PP872793 | **TC-Picorna-LV-237** | 5938nt | par | 10.3158 | MR233-17E/1131 | monocistr., orf-par: prot – RdRp1 – rhv – VP4 – rhv |
| PP872794 | **TC-Picorna-LV-238** | 5932nt | par | 134.817 | MR233-17E/1132 | orf-par: hel – prot – RdRp1 |
| PP872795 | **TC-Picorna-LV-239** | 6107nt | par | 38.7182 | MR233-17E/1140 | orf-par: hel – prot – RdRp1 |
| PP872796 | **TC-Picorna-LV-240** | 5901nt | par | 17.6367 | MR233-17/1143 | orf-par: hel – prot (pfam00548) – RdRp1 |
| PP872797 | **TC-Picorna-LV-241** | 5895nt | par | 65.9518 | MR233-17/1144 | dicistr., orf1-par: prot – RdRp1; orf2-par: rhv – rhv – CRPV |
| PP872798 | **TC-Picorna-LV-242** | 5687nt | par | 88.3357 | MR233-17/1150 | orf-par: hel – prot – RdRp1 |

| | | | | | | |
|---|---|---|---|---|---|---|
| PP872799 | **TC-Picorna-LV-243** | 5874nt | par | 15.5281 | MR233-17E/1157 | dicistr., orf1-par: hel – prot – RdRp1; orf2: - |
| PP872800 | **TC-Picorna-LV-244** | 9134nt | cg | 177.02 | MR233-17E/9134 | dicistr., orf1: hel – prot – RdRp1; orf2: rhv – VP4 – rhv |
| PP872801 | **TC-Picorna-LV-245** | 5826nt | par | 14.9269 | MR233-17E/1175 | orf: hel – prot – RdRp1 |
| PP872802 | **TC-Picorna-LV-246** | 6449nt | par | 83.7707 | MR233-17E/1186 | orf-par: hel – prot – RdRp1 |
| PP872803 | **TC-Picorna-LV-247** | 5770nt | par | 14.008 | MR233-17E/1202 | dicistr., orf1-par: prot (pfam00548) – RdRp1; orf2: rhv – VP4 – rhv |
| PP872804 | **TC-Picorna-LV-248** | 9085nt | cg | 449.04 | MR233-17E/1204 | dicistr., orf1: hel – prot – RdRp1; orf2: rhv – VP4 – rhv |
| PP872805 | **TC-Picorna-LV-249** | 9226nt | cg | 37.5713 | MR233-17E/1205 | dicistr., orf1: hel – prot (pfam00548) – RdRp1; orf2: rhv |
| PP872806 | **TC-Picorna-LV-250** | 5747nt | par | 168.292 | MR233-17E/1213 | orf: hel – prot – RdRp1 |
| PP872807 | **TC-Picorna-LV-251** | 5730nt | par | 18.7209 | MR233-17E/1219 | orf-par: hel – prot – RdRp1 |
| PP872808 | **TC-Picorna-LV-252** | 8509nt | cg | 109.211 | MR233-17E/1239 | dicistr., orf1: hel – prot – RdRp1; orf2: rhv – VP4 – rhv |
| PP872809 | **TC-Picorna-LV-253** | 5669nt | par | 29.6516 | MR233-17E/1247 | monocistr., orf-par: RdRp1 – VP1 – VP2 |
| PP872810 | **TC-Picorna-LV-254** | 5669nt | par | 17.8555 | MR233-17E/1248 | monocistr., orf-par: CRPV – hel – prot – RdRp1 |
| PP872811 | **TC-Picorna-LV-255** | 5935nt | par | 124.43 | MR233-17E/1279 | dicistr., orf1 -par: hel – prot – RdRp1; orf2-par: - |
| PP872812 | **TC-Picorna-LV-256** | 9230nt | cg | 139.061 | MR233-17D/991 | monocistr., orf: hel – prot – RdRp1 – rhv – rhv – rhv |
| PP872813 | **TC-Picorna-LV-257** | 5584nt | par | 9.44878 | MR233-17E/1285 | dicistr., orf1-par: hel – prot – RdRp1; orf2-par: rhv – VP4 |
| PP872814 | **TC-Picorna-LV-258** | 5931nt | par | 13.6385 | MR233-17D/60642 | dicistr., orf1-par: prot – RdRp1; orf2-par: rhv – VP4 – rhv – CRPV |
| PP872815 | **TC-Picorna-LV-259** | 5889nt | par | 15.3974 | MR233-17E/1296 | orf-par: hel – prot – RdRp1 |
| PP872816 | **TC-Picorna-LV-260** | 5497nt | par | 28.624 | MR233-17E/1332 | orf-par: hel – prot (pfam00548) – RdRp1 |
| PP872817 | **TC-Picorna-LV-261** | 7969nt | cg | 58.5055 | MR233-17E/1338 | dicistr., orf1: hel – prot – RdRp1; orf2: rhv – VP4 – rhv |
| PP872818 | **TC-Picorna-LV-262** | 5472nt | par | 22.5735 | MR233-17E/1344 | dicistr., orf1-par: prot – RdRp1 (cd23195; orf2: rhv – VP4 – rhv – CRPV |
| PP872819 | **TC-Picorna-LV-263** | 5395nt | par | 10.2044 | MR233-17E/1382 | monocistr., orf-par: prot (pfam00548) – RdRp1 – rhv – rhv |
| PP872820 | **TC-Picorna-LV-264** | 5385nt | par | 20.8516 | MR233-17E/1390 | orf-par: hel – prot – RdRp1 |
| PP872821 | **TC-Picorna-LV-265** | 9269nt | cg | 70.2933 | MR233-17E/1391 | dicistr., orf1: hel – prot – RdRp1; orf2: rhv – VP4 – rhv |
| PP872822 | **TC-Picorna-LV-266** | 5368nt | par | 39.5378 | MR233-17E/1400 | dicistr., orf: hel – prot – RdRp1 |
| PP872823 | **TC-Picorna-LV-267** | 6013nt | par | 37.3374 | MR233-17E/1418 | dicistr., orf: hel – prot – RdRp1 |
| PP872824 | **TC-Picorna-LV-268** | 9027nt | cg | 62.7197 | MR233-17E/1419 | dicistr., orf1: hel – prot – RdRp1; orf2: rhv – VP4 – rhv – CRPV |
| PP872825 | **TC-Picorna-LV-269** | 5245nt | par | 21.1052 | MR233-17E/1451 | dicistr., orf1-par: prot (pfam00548) – RdRp1; orf2: rhv – rhv |
| PP872826 | **TC-Picorna-LV-270** | 8570nt | cg | 174.658 | MR233-17E/1459 | dicistr., orf1: hel – prot – RdRp1; orf2: rhv – VP4 – rhv |
| PP872827 | **TC-Picorna-LV-271** | 5203nt | par | 112.389 | MR233-17E/1474 | orf: rhv – rhv |
| PP872828 | **TC-Picorna-LV-272** | 5916nt | par | 12.5688 | MR233-17D/54623 | monocistr., orf-par: prot – RdRp1 – rhv – VP4 – rhv |
| PP872829 | **TC-Picorna-LV-273** | 5183nt | par | 16.956 | MR233-17E/5183 | orf-par: hel – prot (pfam12381) – RdRp1 |
| PP872830 | **TC-Picorna-LV-274** | 6820nt | par | 19.0189 | MR233-17E/1512 | orf-par: hel – prot – RdRp1 |
| PP872831 | **TC-Picorna-LV-275** | 5097nt | par | 217.807 | MR233-17E/1538 | dicistr., orf1-par: prot (pfam00548) – RdRp1; orf2-par: rhv – VP4 – rhv – CRPV |
| PP872832 | **TC-Picorna-LV-276** | 5078nt | par | 18.2462 | MR233-17E/1555 | orf-par: hel – prot – RdRp1 |
| PP872833 | **TC-Picorna-LV-277** | 5044nt | par | 21.9348 | MR233-17E/1579 | dicistr., orf1-par: prot – RdRp1; orf2-par: rhv |
| PP872834 | **TC-Picorna-LV-278** | 5177nt | par | 11.5401 | MR233-17E/1601 | orf-par: RdRp1 |
| PP872835 | **TC-Picorna-LV-279** | 4995nt | par | 35.4272 | MR233-17E/1610 | dicistr., orf: hel – prot – RdRp1 |
| PP872836 | **TC-Picorna-LV-280** | 4978nt | par | 19.8558 | MR233-17E/1623 | dicistr., orf1-par: RdRp1; orf2-par: rhv – rhv – CRPV |
| PP872837 | **TC-Picorna-LV-281** | 8407nt | cg | 25.0705 | MR233-17E/1624 | dicistr., orf1: hel – prot – RdRp1 (pfam23195); orf2: rhv – VP4 – rhv – CRPV |
| PP872838 | **TC-Picorna-LV-282** | 4947nt | par | 15.9685 | MR233-17E/1641 | orf-par: hel – prot (pfam00548) – RdRp1 (cd23149) |
| PP872839 | **TC-Picorna-LV-283** | 4938nt | par | 17.181 | MR233-17E/1646 | dicistr., orf1-par: no conserved domains detected, orf2-par: hel |
| PP872840 | **TC-Picorna-LV-284** | 9137nt | cg | 30.0305 | MR233-17E/1652 | dicistr., orf1: hel – prot – RdRp1; orf2: rhv – VP4 – rhv |
| PP872841 | **TC-Picorna-LV-285** | 4928nt | par | 33.6703 | MR233-17E/1653 | dicistr., orf1-par: RdRp1; orf2: rhv – VP4 – rhv |
| PP872842 | **TC-Picorna-LV-286** | 8992nt | cg | 17633.4 | MR233-17E/1659 | dicistr., orf1: hel – prot – RdRp1 (pfam23195); orf2: rhv – VP4 – rhv |
| PP872843 | **TC-Picorna-LV-287** | 5216nt | par | 23.1223 | MR233-17E/1673 | orf-par: hel |
| PP872844 | **TC-Picorna-LV-288** | 4888nt | par | 75.3893 | MR233-17E/1677 | orf: hel – prot – RdRp1 |
| PP872845 | **TV-Picorna-LV-289** | 4882nt | par | 59.7663 | MR233-17E/1679 | orf: hel – prot – RdRp1 |
| PP872846 | **TC-Picorna-LV-290** | 8680nt | cg | 90933.2 | MR233-17E/1687 | dicistr., orf1: hel – prot – RdRp1; orf2: rhv – VP4 – rhv – CRPV |
| PP872847 | **TC-Picorna-LV-291** | 4865nt | par | 11.2164 | MR233-17E/1705 | dicistr., orf1-par: hel – prot – RdRp1; orf2-par: rhv |
| PP872848 | **TC-Picorna-LV-292** | 4848nt | par | 39.9835 | MR233-17E/1714 | dicistr., orf: hel – prot – RdRp1 |
| PP872849 | **TC-Picorna-LV-293** | 4844nt | par | 23.8774 | MR233-17E/1718 | orf-par: hel – prot – RdRp1 |
| PP872850 | **TC-Picorna-LV-294** | 8798nt | cg | 1481.99 | MR233-17D/373 | dicistr., orf1: hel – prot (pfam00548) – RdRp1; orf2: rhv – rhv – CRPV |
| PP872851 | **TC-Picorna-LV-295** | 4801nt | par | 21.4655 | MR233-17E/4801 | 2 orfs, orf1: no conserved domains detected, orf2: hel |
| PP872852 | **TC-Picorna-LV-296** | 4751nt | par | 22.3976 | MR233-17E/4751 | monocistr., orf-par: RdRp – rhv – VP4 – rhv |
| PP872853 | **TC-Picorna-LV-297** | 5117nt | par | 11.5177 | MR233-17E/1858 | unklar, ob mono- oder dicistr., orf-par: hel – prot |
| PP872854 | **TC-Picorna-LV-298** | 7919nt | par | 49.761 | MR233-17E/1887 | monocistr., orf-par: hel – prot – RdRp1 – rhv – VP4 – rhv |
| PP872855 | **TC-Picorna-LV-299** | 2684nt | par | 10.975 | MR233-17E/5440 | monocistr., orf-par: RdRp1 – rhv |
| PP872856 | **TC-Picorna-LV-300** | 4566nt | par | 10.0799 | MR233-17E/1930 | monocistr., orf-par: RdRp1 – rhv – VP4 – rhv |
| PP872857 | **TC-Picorna-LV-301** | 4535nt | par | 14.3943 | MR233-17E/1953 | dicistr., orf1-par: RdRp1; orf2: rhv – rhv -CRPV |
| PP872858 | **TC-Picorna-LV-302** | 4512nt | par | 45.3628 | MR233-17E/1967 | orf-par: hel – prot |
| PP872859 | **TC-Picorna-LV-303** | 9477nt | par | 34.5028 | MR233-17E/1984 | monocistr., orf-par: rhv – hel – prot |
| PP872860 | **TC-Picorna-LV-304** | 8070nt | par | 29.6586 | MR233-17E/1995 | dicistr., orf1: hel – prot – RdRp1; orf2-par: rhv – VP4 – rhv |
| PP872861 | **TC-Picorna-LV-305** | 4473nt | par | 21.2081 | MR233-17E/1996 | dicistr., orf1-par: RdRp1; orf2: rhv – rhv – CRPV capsid |
| PP872862 | **TC-Picorna-LV-306** | 8614nt | cg | 86.2111 | MR233-17E/2004 | dicistr., orf1: hel – prot (pfam00548) – RdRp1; orf2: rhv – VP4 – rhv – CRPV |

| | | | | | | |
|---|---|---|---|---|---|---|
| PP872863 | **TC-Picorna-LV-307** | 5919nt | par | 29.2151 | MR233-17E/2016 | orf-par: hel – prot – RdRp1 – NADAR (cd15457) |
| PP872864 | **TC-Picorna-LV-308** | 8546nt | cg | 24.981 | MR233-17E/2074 | dicistr., orf1: hel – prot – RdRp1; orf2: rhv – rhv |
| PP872865 | **TC-Picorna-LV-309** | 10695nt | cg | 84.9061 | MR233-17E/2079 | dicistr., orf1: (hel) – prot -RdRp1; orf2: rhv – rhv |
| PP872866 | **TC-Picorna-LV-310** | 8031nt | cg | 22.7115 | MR233-17E/2110 | monocistr., orf: rhv – rhv – hel – prot – RdRp1 |
| PP872867 | **TC-Picorna-LV-311** | 4524nt | par | 13.3627 | MR233-17E/2126 | dicistr., orf1-par: RdRp1; orf2: rhv – VP4 – rhv – CRPV capsid |
| PP872868 | **TC-Picorna-LV-312** | 4306nt | par | 13.1359 | MR233-17E/2151 | orf-par: hel – prot |
| PP872869 | **TC-Picorna-LV-313** | 8355nt | cg | 44.2878 | MR233-17E/2153 | dicistr., orf1: hel – prot – RdRp1; orf2: rhv – rhv – CRPV |
| PP872870 | **TC-Picorna-LV-314** | 4296nt | par | 15.2037 | MR233-17E/2168 | dicistr., orf1-par: RdRp1; orf2: rhv – VP4 – rhv |
| PP872871 | **TC-Picorna-LV-315** | 4295nt | par | 11.98 | MR233-17E/2169 | dicistr., orf1-par: prot – RdRp1; orf2-par: rhv – VP4 – rhv |
| PP872872 | **TC-Picorna-LV-316** | 4290nt | par | 20.2359 | MR233-17E/2171 | monocistr., orf-par: RdRp1 – rhv – rhv |
| PP872873 | **TC-Picorna-LV-317** | 2835nt | par | 48.0374 | MR233-17E/5442 | orf-par: hel |
| PP872874 | **TC-Picorna-LV-318** | 3392nt | par | 10.6863 | MR233-17E/2201 | orf-par: hel |
| PP872875 | **TC-Picorna-LV-319** | 6060nt | par | 18.0924 | MR233-17E/2208 | dicistr., orf1-par: hel – prot – RdRp1; orf2-par: – |
| PP872876 | **TC-Picorna-LV-320** | 4247nt | par | 19.3042 | MR233-17E/2219 | dicistr., orf1-par: RdRp1; orf2: rhv – VP4 – rhv |
| PP872877 | **TC-Picorna-LV-321** | 2710nt | par | 20.0461 | MR233-17E/5342 | dicistr., orf: rhv – VP4 – rhv – CRPV |
| PP872878 | **TC-Picorna-LV-322** | 9013nt | cg | 1203.24 | MR233-17E/2232 | monocistr., orf: hel – prot (pfam00548) – RdRp – rhv -rhv |
| PP872879 | **TC-Picorna-LV-323** | 4230nt | par | 17.4248 | MR233-17E/2234 | dicistr., orf1-par: RdRp1; orf2: rhv – VP4 – rhv |
| PP872880 | **TC-Picorna-LV-324** | 4212nt | par | 12.3545 | MR233-17E/2251 | dicistr., orf1-par: RdRp1; orf2-par: rhv – VP4 – rhv – CRPV |
| PP872881 | **TC-Picorna-LV-325** | 3350nt | par | 22.209 | MR233-17E/5508 | orf-par: hel |
| PP872882 | **TC-Picorna-LV-326** | 7895nt | cg | 686.593 | MR233-17E/2263 | dicistr., orf1: hel – prot (pfam00548) – RdRp1; orf2: rhv – VP4 – rhv – CRPV |
| PP872883 | **TC-Picorna-LV-327** | 4326nt | par | 42.5724 | MR233-17E/2265 | orf-par: hel – prot (pfam00548) – RdRp1 |
| PP872884 | **TC-Picorna-LV-328** | 4186nt | par | 13.4835 | MR233-17E/2273 | dicistr., orf1: rhv – rhv – CRPV; orf2-par: – |
| PP872885 | **TC-Picorna-LV-329** | 5408nt | par | 15.3835 | MR233-17E/57915 | monocistr., orf-par: hel – prot – RdRp1 – rhv – VP4 – rhv |
| PP872886 | **TC-Picorna-LV-330** | 5130nt | par | 41.8546 | MR233-17E/2280 | dicistr., orf1-par: prot (pfam00548) – RdRp1; orf2: rhv |
| PP872887 | **TC-Picorna-LV-331** | 4177nt | par | 7.63299 | MR233-17E/2285 | orf-par: hel – prot |
| PP872888 | **TC-Picorna-LV-332** | 8373nt | cg | 56.8174 | MR233-17E/2306 | dicistr., orf1: hel – prot (pfam00548) – RdRp1; orf2: rhv – VP4 – rhv – CRPV |
| PP872889 | **TC-Picorna-LV-333** | 4145nt | par | 16.6456 | MR233-17E/2320 | unklar, ob mono- oder dicistr., orf-par: hel – prot – RdRp1 |
| PP872890 | **TC-Picorna-LV-334** | 4133nt | par | 11.5437 | MR233-17E/2330 | dicistr., orf1-par: prot (pfam00548) – RdRp1; orf2-par: rhv – rhv |
| PP872891 | **TC-Picorna-LV-335** | 9214nt | cg | 5787.72 | MR233-17E/2332 | dicistr., orf1: hel – prot – RdRp1; orf2: rhv – VP4 – rhv |
| PP872892 | **TC-Picorna-LV-336** | 4130nt | par | 11.9692 | MR233-17E/2337 | orf-par: hel – prot – RdRp1 |
| PP872893 | **TC-Picorna-LV-337** | 4121nt | par | 16.6117 | MR233-17E/2343 | dicistr., orf1-par: prot – RdRp1 – NADAR (cd15457); orf2-par: rhv |
| PP872894 | **TC-Picorna-LV-338** | 4097nt | par | 22.5907 | MR233-17E/2363 | orf-par: no conserved domains detected |
| PP872895 | **TC-Picorna-LV-339** | 4094nt | par | 13.1549 | MR233-17E/2368 | dicistr., orf-par: –; orf2-par: rhv – Dicistr VP4 – rhv – rhv |
| PP872896 | **TC-Picorna-LV-340** | 4097nt | par | 17.6699 | MR233-17E/2378 | orf-par: hel – prot |
| PP872897 | **TC-Picorna-LV-341** | 4085nt | par | 13.1621 | MR233-17E/2380 | orf-par: hel – prot – RdRp1 (cd23193) |
| PP872898 | **TC-Picorna-LV-342** | 4075nt | par | 13.0709 | MR233-17E/2395 | orf-par: hel |
| PP872899 | **TC-Picorna-LV-343** | 4063nt | par | 15.2481 | MR233-17E/2412 | dicistr., orf1-par: RdRp1, orf2-par: rhv – rhv |
| PP872900 | **TC-Picorna-LV-344** | 4790nt | par | 11.3253 | MR233-17E/2428 | monocistr, orf-par: hel – prot – RdRp1 – rhv |
| PP872901 | **TC-Picorna-LV-345** | 4046nt | par | 18.6406 | MR233-17E/2431 | dicistr., orf1-par: RdRp1; orf2: rhv – VP4 – rhv |
| PP871902 | **TC-Picorna-LV-346** | 4084nt | par | 61.8763 | MR233-17E/2436 | orf-par: hel |
| PP872903 | **TC-Picorna-LV-347** | 3379nt | par | 11.8778 | MR233-17E/3538 | orf-par: prot – RdRp1 |
| PP872904 | **TC-Picorna-LV-348** | 3963nt | par | 14.7908 | MR233-17E/2532 | orf-par: hel – prot – RdRp1 |
| PP872905 | **TC-Picorna-LV-349** | 3952nt | par | 11.6364 | MR233-17E/2547 | monocistr., orf-par: RdRp1 – rhv – rhv – CRPV |
| PP872906 | **TC-Picorna-LV-350** | 3749nt | par | 25.8962 | MR233-17E/2547 | orf-par: hel – prot |
| PP872907 | **TC-Picorna-LV-351** | 3908nt | par | 65.3608 | MR233-17E/2616 | 4 orfs, orf1: –; orf2: –; orf3: –; orf4: – |
| PP872908 | **TC-Picorna-LV-352** | 4537nt | par | 13.7642 | MR233-17E/2626 | dicistr., orf1-par: prot (pfam00548) – RdRp1; orf2-par: rhv – VP4 – rhv – CRPV |
| PP873909 | **TC-Picorna-LV-353** | 9666nt | cg | 191.094 | MR233-17D/1567 | monocistr., orf: hel – prot – RdRp1 – rhv – rhv |
| PP872910 | **TC-Picorna-LV-354** | 4221nt | par | 21.1111 | MR233-17E/2664 | dicistr., orf1-par: prot – RdRp1; orf2-par: rhv – rhv |
| PP872911 | **TC-Picorna-LV-355** | 3851nt | par | 10.8367 | MR233-17E/2694 | orf-par: hel – prot |
| PP872912 | **TC-Picorna-LV-356** | 3849nt | par | 11.1029 | MR233-17E/2699 | monocistr., orf-par: prot – RdRp1 – rhv – VP4 |
| PP872913 | **TC-Picorna-LV-357** | 3832nt | par | 7.94442 | MR233-17E/2720 | orf-par: RdRp1 |
| PP872914 | **TC-Picorna-LV-358** | 3805nt | par | 152.501 | MR233-17E/2766 | orf-par: hel |
| PP872915 | **TC-Picorna-LV-359** | 3796nt | par | 9.49499 | MR233-17E/2780 | dicistr., orf1-par: -, orf2-par: hel |
| PP872916 | **TC-Picorna-LV-360** | 3788nt | par | 26.2381 | MR233-17E/2790 | dicistr., orf1-par: RdRp1, orf2: rhv – VP4 – rhv |
| PP872917 | **TC-Picorna-LV-361** | 3843nt | par | 13.0809 | MR233-17E/2791 | orf-par: hel – prot |
| PP872918 | **TC-Picorna-LV-362** | 3371nt | par | 20.1643 | MR233-17E/3552 | dicistr., orf1-par: –; orf2: rhv – rhv |
| PP872919 | **TC-Picorna-LV-363** | 3747nt | par | 18.3437 | MR233-17E/2845 | orf-par: – |
| PP872920 | **TC-Picorna-LV-364** | 3742nt | par | 16.2167 | MR233-17E/2856 | dicistr., orf1-par: prot – RdRp1; orf2-par: – |
| PP872921 | **TC-Picorna-LV-365** | 4167nt | par | 18.2755 | MR233-17E/2863 | orf-par: hel – prot |
| PP872922 | **TC-Picorna-LV-366** | 3737nt | par | 10.8124 | MR233-17E/2864 | dicistr., orf1-par: RdRp1; orf2-par: rhv – VP4 – rhv – CRPV |
| PP872923 | **TC-Picorna-LV-367** | 3727nt | par | 13.15 | MR233-17E/2878 | orf-par: hel – prot – RdRp1 |
| PP872824 | **TC-Picorna-LV-368** | 3370nt | par | 20.8395 | MR233-17E/3555 | orf-par: hel |
| PP872925 | **TC-Picorna-LV-369** | 3724nt | par | 11.9272 | MR233-17E/2883 | orf-par: hel – prot (pfam00548) |
| PP872926 | **TC-Picorna-LV-370** | 3352nt | par | 20.1023 | MR233-17E/3577 | orf-par: hel |

Table S1

| | | | | | | |
|---|---|---|---|---|---|---|
| PP872927 | **TC-Picorna-LV-371** | 5764nt | par | 18.7327 | MR233-17E/2911 | dicistr., orf1-par: prot – RdRp1; orf2: rhv – VP4 – rhv |
| PP872928 | **TC-Picorna-LV-372** | 3702nt | par | 13.802 | MR233-17E/2931 | dicistr., orf1-par: RdRp1; orf2-par: rhv – VP4 – rhv |
| PP872929 | **TC-Picorna-LV-373** | 5515nt | par | 19.398 | MR233-17E/28527 | dicistr., orf1-par: prot – RdRp1; orf2: rhv – VP4 – rhv |
| PP872930 | **TC-Picorna-LV-374** | 3687nt | par | 28.7673 | MR233-17E/2954 | orf-par: rhv – VP4 – rhv – CRPV |
| PP872931 | **TC-Picorna-LV-375** | 4699nt | par | 46.3769 | MR233-17E/2958 | orf-par: hel – prot (pfam00548) -RdRp1 |
| PP872932 | **TC-Picorna-LV-376** | 3345nt | par | 8.76263 | MR233-17E/3596 | dicistr., orf1-par: RdRp1; orf2-par: rhv – VP4 – rhv |
| PP872933 | **TC-Picorna-LV-377** | 3662nt | par | 22.84 | MR233-17E/2994 | dicistr., orf1: RdRp1; orf2-par: rhv – VP4 – rhv |
| PP872934 | **TC-Picorna-LV-378** | 8704nt | cg | 141.696 | MR233-17E/3020 | monocistr., orf: hel – prot – RdRp1 – rhv – rhv – rhv |
| PP872935 | **TC-Picorna-LV-379** | 3633nt | par | 12.6083 | MR233-17E/3041 | dicistr., orf1-par: RdRp1; orf2-par: rhv – VP4 – rhv |
| PP872936 | **TC-Picorna-LV-380** | 7684nt | par | 97.9231 | MR233-17E/3048 | monocistr., orf-par: hel – prot – RdRp1 – rhv – VP4 – rhv |
| PP872937 | **TC-Picorna-LV-381** | 3603nt | par | 8.92367 | MR233-17E/3098 | orf-par: hel |
| PP872938 | **TC-Picorna-LV-382** | 3338nt | par | 16.8694 | MR233-17E/3606 | orf-par: hel – prot (pfam00548) – RdRp1 |
| PP872939 | **TC-Picorna-LV-383** | 3786nt | par | 13.4614 | MR233-17E/3105 | orf-par: hel – prot – RdRp1 |
| PP872940 | **TC-Picorna-LV-384** | 4592nt | par | 25.3493 | MR233-17E/3652 | orf-par: hel – prot |
| PP872941 | **TC-Picorna-LV-385** | 3578nt | par | 11.6987 | MR233-17E/3142 | dicistr., orf1-par: prot (pfam00548) – RdRp1; orf2-par: rhv |
| PP872942 | **TC-Picorna-LV-386** | 8479nt | cg | 189.728 | MR233-17E/3170 | dicistr., orf1: hel – prot – RdRp1; orf2-par: rhv – VP4 – rhv |
| PP872943 | **TC-Picorna-LV-387** | 3555nt | par | 8.0993 | MR233-17E/3184 | orf-par: hel – prot (pfam00548) – RdRp1 |
| PP872944 | **TC-Picorna-LV-388** | 7569nt | par | 20.833 | MR233-17E/3199 | orf-par: hel – prot – RdRp1 |
| PP872945 | **TC-Picorna-LV-389** | 3531nt | par | 8.98641 | MR233-17E/3240 | dicistr., orf1-par: prot (pfam00548) – RdRp1; orf2-par: – |
| PP872946 | **TC-Picorna-LV-390** | 3531nt | par | 7.91306 | MR233-17E/3241 | orf-par: hel – prot – RdRp1 |
| PP872947 | **TC-Picorna-LV-391** | 3664nt | par | 21.0109 | MR233-17E/3257 | orf-par: hel |
| PP872948 | **TC-Picorna-LV-392** | 3515nt | par | 22.159 | MR233-17E/3269 | orf-par: hel |
| PP872949 | **TC-Picorna-LV-393** | 5512nt | par | 20.9822 | MR233-17E/3679 | orf-par: hel – prot (pfam12381) – RdRp1 |
| PP872950 | **TC-Picorna-LV-394** | 3475nt | par | 14.8043 | MR233-17E/3341 | dicistr., orf1-par: RdRp1; orf2-par: rhv |
| PP872951 | **TC-Picorna-LV-395** | 3456nt | par | 7.27315 | MR233-17E/3456 | orf-par: hel |
| PP872952 | **TC-Picorna-LV-396** | 3642nt | par | 7.76936 | MR233-17E/3391 | dicistr., orf1-par: -, orf2: rhv – rhv |
| PP872953 | **TC-Picorna-LV-397** | 3443nt | par | 11.2533 | MR233-17E/3418 | monocistr., orf-par: RdRp1 – rhv – VP4 – rhv |
| PP872954 | **TC-Picorna-LV-398** | 3430nt | par | 9.91924 | MR233-17E/3439 | dicistr., orf1-par: RdRp1 (23195); orf2-par: rhv – VP4 – rhv |
| PP872955 | **TC-Picorna-LV-399** | 5597nt | par | 856.903 | MR233-17E/3683 | orf-par: hel – prot – RdRp1 (cd23210) |
| PP872956 | **TC-Picorna-LV-400** | 3309nt | par | 10.6035 | MR233-17E/3685 | orf-par: hel – prot |
| PP872957 | **TC-Picorna-LV-401** | 5949nt | par | 94.3137 | MR233-17E/3694 | orf-par: hel – prot – RdRp1 |
| PP872958 | **TC-Picorna-LV-402** | 8390nt | cg | 73.5365 | MR233-17E/3701 | dicistr., orf1: hel – prot (pfam00548) – RdRp1; orf2-par: rhv |
| PP872959 | **TC-Picorna-LV-403** | 6738nt | par | 11.5518 | MR233-17E/3716 | monocistr., orf-par: prot – RdRp1 – rhv – rhv |
| PP872960 | **TC-Picorna-LV-404** | 4044nt | par | 18.3583 | MR233-17E/4077 | monocistr., orf-par: RdRp1 – rhv – VP4 – rhv |
| PP872961 | **TC-Picorna-LV-405** | 3740nt | par | 14.2783 | MR233-17E/3718 | orf-par: rhv – rhv – CRPV |
| PP872962 | **TC-Picorna-LV-406** | 3287nt | par | 11.0636 | MR233-17E/3734 | dicistr., orf1-par: RdRp1; orf2-par: rhv – VP4 – rhv – CRPV |
| PP872963 | **TC-Picorna-LV-407** | 5442nt | par | 30.3282 | MR233-17E/3763 | monocistr., orf-par: RdRp1 – rhv – rhv |
| PP872964 | **TC-Picorna-LV-408** | 3264nt | par | 19.9078 | MR233-17E/3779 | orf-par: rhv – rhv |
| PP872965 | **TC-Picorna-LV-409** | 3252nt | par | 13.131 | MR233-17E/3805 | orf-par: rhv – rhv – CRPV |
| PP872966 | **TC-Picorna-LV-410** | 3245nt | par | 10.8404 | MR233-17E/3820 | orf-par: hel |
| PP873967 | **TC-Picorna-LV-411** | 8057nt | par | 1016.7 | MR233-17E/3821 | orf-par: hel – prot – RdRp1 |
| PP872968 | **TC-Picorna-LV-412** | 3130nt | par | 15.3629 | MR233-17E/3832 | orf-par: hel |
| PP872969 | **TC-Picorna-LV-413** | 3224nt | par | 38.4634 | MR233-17E/3869 | dicistr., orf: rhv – VP4 – rhv – CRPV |
| PP872970 | **TC-Picorna-LV-414** | 3223nt | par | 8.29134 | MR233-17E/3870 | orf-par: rhv – rhv |
| PP872971 | **TC-Picorna-LV-415** | 3220nt | par | 18.1593 | MR233-17E/3878 | orf-par: rhv – rhv |
| PP872972 | **TC-Picorna-LV-416** | 3218nt | par | 8.76259 | MR233-17E/3881 | dicistr., orf1-par: RdRp1; orf2-par: rhv |
| PP872973 | **TC-Picorna-LV-417** | 3215nt | par | 47.5054 | MR233-17E/3887 | dicistr., orf: rhv – rhv |
| PP872974 | **TC-Picorna-LV-418** | 3365nt | par | 8.42615 | MR233-17E/3889 | monocistr., orf-par: RdRp1 – rhv – VP4 – rhv |
| PP872975 | **TC-Picorna-LV-419** | 9015nt | cg | 111.498 | MR233-17E/3891 | dicistr., orf1: hel – prot – RdRp1; orf2: rhv – VP4 – rhv |
| PP872976 | **TC-Picorna-LV-420** | 3212nt | par | 22.4328 | MR233-17E/3895 | monocistr., orf-par: RdRp1 – rhv – rhv – CRPV |
| PP872977 | **TC-Picorna-LV-421** | 5922nt | par | 72.1999 | MR233-17E/3896 | dicistr., orf: hel – prot – RdRp1 |
| PP872978 | **TC-Picorna-LV-422** | 3209nt | par | 7.85011 | MR233-17E/3900 | orf-par: hel |
| PP872979 | **TC-Picorna-LV-423** | 3197nt | par | 51.97 | MR233-17E/3927 | orf-par: rhv – VP4 – rhv |
| PP872980 | **TC-Picorna-LV-424** | 11573nt | cg | 55.4267 | MR233-17E/3980 | 5 orfs, orf1,2,3: –; orf4: prot (cd20171); orf5: hel – RdRp1 |
| PP872981 | **TC-Picorna-LV-425** | 3172nt | par | 19.5646 | MR233-17E/3982 | dicistr., orf1-par: prot – RdRp1 (cd23200); orf2-par: – |
| PP872982 | **TC-Picorna-LV-426** | 4945nt | par | 16.0413 | MR233-17E/3983 | orf-par: hel – prot – RdRp1 |
| PP872983 | **TC-Picorna-LV-427** | 3166nt | par | 7.07265 | MR233-17E/3995 | orf-par: hel – prot (pfam00548) – RdRp1 |
| PP872984 | **TC-Picorna-LV-428** | 3164nt | par | 63.2475 | MR233-17E/4000 | orf-par: RdRp1 |
| PP872985 | **TC-Picorna-LV-429** | 3161nt | par | 22.906 | MR233-17E/4006 | dicistr., orf1-par: RdRp1; orf2: rhv – rhv – CRPV |
| PP872986 | **TC-Picorna-LV-430** | 3160nt | par | 9.77816 | MR233-17E/4011 | orf-par: prot – RdRp1 |
| PP872987 | **TC-Picorna-LV-431** | 4669nt | par | 15.4099 | MR233-17E/4050 | dicistr., orf1-par: RdRp1; orf2: rhv – VP4 – rhv |
| PP872988 | **TC-Picorna-LV-432** | 3343nt | par | 9.41789 | MR233-17E/4065 | orf-par: rhv – rhv – CRPV |
| PP872989 | **TC-Picorna-LV-433** | 3127nt | par | 21.487 | MR233-17E/4085 | orf-par: hel |
| PP872990 | **TC-Picorna-LV-434** | 3127nt | par | 16.3518 | MR233-17E/4086 | monocistr., orf-par: RdRp1 – rhv – VP4 – rhv |

| | | | | | | |
|---|---|---|---|---|---|---|
| PP872991 | **TC-Picorna-LV-435** | 3119nt | par | 9.24559 | MR233-17E/4111 | monocistr., orf-par: RdRp1 – rhv – rhv |
| PP872992 | **TC-Picorna-LV-436** | 3197nt | par | 24.0766 | MR233-17E/4115 | orf-par: hel |
| PP872993 | **TC-Picorna-LV-437** | 3115nt | par | 9.45265 | MR233-17E/4124 | dicistr., orf1-par: RdRp1; orf2-par: rhv – VP4 – rhv |
| PP872994 | **TC-Picorna-LV-438** | 4786nt | par | 9.08734 | MR233-17E/4129 | dicistr., orf1-par: prot (pfam12381) – RdRp1; orf2-par: rhv – VP4 – rhv – CRPV |
| PP872995 | **TC-Picorna-LV-439** | 3095nt | par | 11.0397 | MR233-17E/4165 | orf-par: rhv – VP4 – rhv |
| PP872996 | **TC-Picorna-LV-440** | 3090nt | par | 14.1333 | MR233-17E/4177 | orf-par: rhv |
| PP872997 | **TC-Picorna-LV-441** | 3285nt | par | 114.657 | MR233-17E/4185 | orf-par: hel |
| PP872998 | **TC-Picorna-LV-442** | 4627nt | par | 15.3972 | MR233-17E/4208 | dicistr., orf1-par: prot (pfam00548) – RdRp1; orf2-par: rhv – VP4 – rhv |
| PP872999 | **TC-Picorna-LV-443** | 3363nt | par | 13.8213 | MR233-17E/4209 | orf-par: rhv – rhv |
| PP873000 | **TC-Picorna-LV-444** | 8637nt | cg | 116.898 | MR233-17E/5370 | dicistr., orf1: hel – prot – RdRp1; orf2: rhv – VP4 – rhv |
| PP873001 | **TC-Picorna-LV-445** | 3071nt | par | 11.2862 | MR233-17E/4220 | orf-par: hel |
| PP873002 | **TC-Picorna-LV-446** | 3054nt | par | 9.95285 | MR233-17E/4265 | orf-par: hel |
| PP873003 | **TC-Picorna-LV-447** | 3051nt | par | 8.89217 | MR233-17E/4272 | orf-par: rhv – VP4 – rhv |
| PP873004 | **TC-Picorna-LV-448** | 3040nt | par | 14.3322 | MR233-17E/4291 | orf-par: rhv – rhv |
| PP873005 | **TC-Picorna-LV-449** | 5730nt | par | 330.377 | MR233-17E/4310 | orf-par: hel – prot – RdRp1 |
| PP873006 | **TC-Picorna-LV-450** | 3004nt | par | 9.09021 | MR233-17E/4381 | orf-par: prot – RdRp1 |
| PP873007 | **TC-Picorna-LV-451** | 3003nt | par | 11.7865 | MR233-17E/4384 | orf-par: RdRp1 |
| PP873008 | **TC-Picorna-LV-452** | 2993nt | par | 13.8466 | MR233-17E/4408 | orf-par: rhv – rhv |
| PP873009 | **TC-Picorna-LV-453** | 5622nt | par | 407.744 | MR233-17E/59492 | orf-par: hel – prot – RdRp1 |
| PP873010 | **TC-Picorna-LV-454** | 3800nt | par | 49.6532 | MR233-17E/2773 | 4 orfs, orf1: –, orf2: –, orf3: –, orf4: – |
| PP873011 | **TC-Picorna-LV-455** | 5757nt | par | 15.8741 | MR233-17E/4487 | orf-par: hel – prot – RdRp1 |
| PP873012 | **TC-Picorna-LV-456** | 2968nt | par | 10.6402 | MR233-17E/4488 | orf-par: hel |
| PP873013 | **TC-Picorna-LV-457** | 3094nt | par | 7.57304 | MR233-17E/4489 | orf-par: rhv – VP4 – rhv |
| PP873014 | **TC-Picorna-LV-458** | 2956nt | par | 9.13329 | MR233-17E/4514 | orf-par: hel |
| PP873015 | **TC-Picorna-LV-459** | 2951nt | par | 13.6225 | MR233-17E/4526 | orf-par: prot (pfam12381) – RdRp1 |
| PP873016 | **TC-Picorna-LV-460** | 2929nt | par | 126.207 | MR233-17E/4585 | orf-par: rhv – VP4 – rhv – CRPV |
| PP873017 | **TC-Picorna-LV-461** | 5292nt | par | 36.3951 | MR233-17E/4594 | dicistr., orf1-par: hel – prot – RdRp1; orf2-par: rhv – rhv |
| PP873018 | **TC-Picorna-LV-462** | 3939nt | par | 12.2249 | MR233-17E/4635 | orf-par: hel |
| PP873019 | **TC-Picorna-LV-463** | 3272nt | par | 30.7809 | MR233-17E/4687 | orf-par: rhv – rhv – CRPV |
| PP873020 | **TC-Picorna-LV-464** | 2890nt | par | 18.682 | MR233-17E/4694 | orf-par: rhv – VP4 – rhv |
| PP873021 | **TC-Picorna-LV-465** | 2888nt | par | 11.5194 | MR233-17E/4700 | orf-par: rhv – VP4 – rhv |
| PP873022 | **TC-Picorna-LV-466** | 2714nt | par | 15.5582 | MR233-17E/5321 | orf-par: rhv – VP4 – rhv |
| PP873023 | **TC-Picorna-LV-467** | 3454nt | par | 296.633 | MR233-17E/4744 | dicistr., orf1-par: prot – RdRp1; orf2-par: rhv |
| PP873024 | **TC-Picorna-LV-468** | 2860nt | par | 10.6434 | MR233-17E/4774 | orf-par: hel |
| PP873025 | **TC-Picorna-LV-469** | 2858nt | par | 21.2792 | MR233-17E/4781 | dicistr., orf: rhv – VP4 – rhv – CRPV |
| PP873026 | **TC-Picorna-LV-470** | 2854nt | par | 18.054 | MR233-17E/4804 | dicistr., orf: rhv – rhv – CRPV |
| PP873027 | **TC-Picorna-LV-471** | 2852nt | par | 14.3236 | MR233-17E/4813 | orf-par: hel |
| PP873028 | **TC-Picorna-LV-472** | 2851nt | par | 13.819 | MR233-17E/4816 | orf-par: prot – RdRp1 |
| PP873029 | **TC-Picorna-LV-473** | 2840nt | par | 21.6123 | MR233-17E/4855 | orf-par: rhv – rhv |
| PP873030 | **TC-Picorna-LV-474** | 2837nt | par | 15.2626 | MR233-17E/4865 | orf-par: hel – prot |
| PP873031 | **TC-Picorna-LV-475** | 2832nt | par | 306.89 | MR233-17E/4880 | orf-par: rhv – VP4 – rhv – CRPV |
| PP873032 | **TC-Picorna-LV-476** | 2831nt | par | 21.1992 | MR233-17E/4887 | orf-par: hel |
| PP873033 | **TC-Picorna-LV-477** | 2830nt | par | 27.9767 | MR233-17E/4889 | orf-par: hel |
| PP873034 | **TC-Picorna-LV-478** | 3168nt | par | 21.542 | MR233-17E/4890 | dicistr., orf1-par: RdRp1; orf2-par: rhv – rhv |
| PP873035 | **TC-Picorna-LV-479** | 3381nt | par | 10.5682 | MR233-17E/4900 | dicistr., orf1-par: RdRp1; orf2: rhv – VP4 – rhv |
| PP873036 | **TC-Picorna-LV-480** | 2807nt | par | 19.932 | MR233-17E/4973 | orf-par: prot – RdRp1 |
| PP873037 | **TC-Picorna-LV-481** | 2880nt | par | 20.3757 | MR233-17E/4985 | dicistr., orf1-par: RdRp1; orf2-par: rhv |
| PP873038 | **TC-Picorna-LV-482** | 2800nt | par | 10.9379 | MR233-17E/4999 | orf-par: rhv |
| PP873039 | **TC-Picorna-LV-483** | 3324nt | par | 13.2136 | MR233-17E/5008 | dicistr., orf1-par: –; orf2: rhv – VP4 – rhv |
| PP873040 | **TC-Picorna-LV-484** | 2790nt | par | 13.9237 | MR233-17E/5030 | 2 orfs, orf1: hel; orf2: – |
| PP873041 | **TC-Picorna-LV-485** | 5149nt | par | 144.628 | MR233-17E/5054 | orf1-par: RdRp1; orf2: rhv – rhv |
| PP873042 | **TC-Picorna-LV-486** | 2715nt | par | 9.35727 | MR233-17E/5320 | orf-par: hel |
| PP873043 | **TC-Picorna-LV-487** | 2762nt | par | 22.2484 | MR233-17E/5131 | orf-par: rhv – VP4 – rhv |
| PP873044 | **TC-Picorna-LV-488** | 7784nt | par | 729.072 | MR233-17E/5144 | dicistr., orf1-par: hel – prot – RdRp1; orf2-par: rhv – rhv |
| PP873045 | **TC-Picorna-LV-489** | 2969nt | par | 7.83159 | MR233-17E/5159 | monocistr., orf-par: RdRp1 – rhv – rhv – CRPV |
| PP873046 | **TC-Picorna-LV-490** | 2753nt | par | 33.6873 | MR233-17E/5168 | orf-par: rhv – VP4 – rhv |
| PP873047 | **TC-Picorna-LV-491** | 3382nt | par | 8.20816 | MR233-17E/5190 | dicistr., orf1-par: RdRp1; orf2-par: rhv -VP4 – rhv |
| PP873048 | **TC-Picorna-LV-492** | 2745nt | par | 8.48452 | MR233-17E/5193 | dicistr., orf1-par: RdRp1; orf2-par: rhv – VP4 – rhv – CRPV |
| PP873049 | **TC-Picorna-LV-493** | 4148nt | par | 19.9547 | MR233-17E/5206 | dicistr., orf1-par: RdRp1; orf2: rhv – rhv – CRPV |
| PP873050 | **TC-Picorna-LV-494** | 3446nt | par | 10.9663 | MR233-17E/5222 | orf-par: hel – prot – RdRp1 |
| PP873051 | **TC-Picorna-LV-495** | 3546nt | par | 11.5917 | MR233-17E/5255 | orf-par: rhv – VP4 – rhv – CRPV |
| PP873052 | **TC-Picorna-LV-496** | 3987nt | par | 8.1856 | MR233-17E/5280 | orf-par: hel – prot – RdRp1 |
| PP873053 | **TC-Picorna-LV-497** | 2722nt | par | 8.30051 | MR233-17E/5295 | orf-par: prot (pfam00548) – RdRp1 |
| PP873054 | **TC-Picorna-LV-498** | 4328nt | par | 1166.07 | MR233-17E/5316 | dicistr., orf1-par: RdRp1; orf2-par: rhv – rhv – CRPV |

Table S1

| | | | | | | |
|---|---|---|---|---|---|---|
| PP873055 | **TC-Picorna-LV-499** | 5133nt | par | 818.973 | MR233-17E/5335 | dicistr., orf1-par: RdRp1; orf2-par: rhv – rhv – CRPV |
| PP873056 | **TC-Picorna-LV-500** | 2710nt | par | 11.3192 | MR233-17E/5345 | dicistr., orf1-par: RdRp1; orf2-par: rhv |
| PP873057 | **TC-Picorna-LV-501** | 2054nt | par | 7.80477 | MR233-17E/9009 | orf-par: RdRp1 |

Abbreviations: cg, complete genome; par, partial genome; hel, helicase; prot, proteinase; rhv, capsid protein with similarity to rhinovirus capsid protein; CRPV, capsid protein with similarity to dicistrovirus capsid protein; VP4, capsid protein with similarity to dicistrovirus VP4 capsid protein; RdRp1, RNA-dependent RNA polymerase superfamily 1
